# Marine Microorganism-Derived Macrolactins Inhibit Inflammatory Mediator Effects in LPS-Induced Macrophage and Microglial Cells by Regulating BACH1 and HO-1/Nrf2 Signals through Inhibition of TLR4 Activation

**DOI:** 10.3390/molecules25030656

**Published:** 2020-02-04

**Authors:** Eun-Nam Kim, Ming Gao, Hyukjae Choi, Gil-Saeng Jeong

**Affiliations:** 1 College of Pharmacy, Keimyung University, 1095 Dalgubeol-daero, Daegu 42601, Korea; enkimpharm@gmail.com; 2 College of Pharmacy, Yeungnam University, 280 Daehak-ro, Gyeongsan 38541, Korea; gaoming104@hotmail.com

**Keywords:** heme oxygenase (HO)-1, nuclear factor erythroid 2-related factor 2 (Nrf2), BTB Domain and CNC Homolog 1 (BACH1), Toll-like receptor 4 (TLR4)

## Abstract

Recently, many natural products with unique structure and promising pharmacological potential have been reported from marine-derived microorganisms. The macrolactin A (MA), 15-*epi*-dihydromacrolactin F (DMF) and macrolactin F (MF) were obtained from the culture broth extract of a marine sediment derived microorganism *Bacillus* sp. HC001. In this study, MA, DMF and MF inhibited the production and expression of proinflammatory mediators of inducible nitric oxide synthase (iNOS) and cyclooxygenase–2 (COX-2) in LPS-stimulated RAW264.7 and BV2 cells. Also, MA, DMF and MF exert anti-inflammatory effects through the expression of heme oxygenase (HO) -1, a stress-inducing enzyme that converts heme to carbon monoxide (CO), iron and biliberdine. Toll-like receptor 4 (TLR4) expressed by lipopolysaccharide (LPS) was inhibited by increased expression of HO-1 transcription factor Nrf2 and down regulation of BTB Domain And CNC Homolog 1 (BACH1), inhibited phosphorylation of Mitogen-activated protein kinase kinase kinase 7 (MAP3K7, TAK1) and nuclear factor kappaB (NF-κB). These results show that MA, DMF and MF effectively inhibited TLR4 by regulating BACH1 and HO-1/Nrf2 signals in LPS-stimulated RAW264.7 and BV2 cells, which suggests the possibility of use as an anti-inflammatory agent.

## 1. Introduction

Inflammation is the initial bodys defense response to tissue damage and foreign pathogens, which protects and regenerates tissue in response to damage and infection, while chronic inflammation results in loss of the immune system, resulting in tissue damage and the development of various diseases [1,2]. In the innate immune response, macrophages and microglia serve as the bodys first line of defense against pathogen invasion and play an important role in promoting cellular protection and repair [3,4]. The activation of macrophages and microglia plays an important role in host defense responses to infectious agents by releasing inflammatory cytokine tumor necrosis factor-alpha (TNF-α), interleukin and pro-inflammatory mediators nitric oxide (NO) [5,6]. Toll-like receptor 4 (TLR4) is a transmembrane protein of pattern recognition receptors for Lipopolysaccharides (LPS) from Gram-positive bacteria that controls early inflammatory responses and plays an important role in inducing the innate immune system [7]. TLR4 activated by LPS activates mitogen-activated protein kinase (MAPK) and nuclear factor B (NF-κB) to induce the production of inflammatory mediators and expresses inflammatory factors including matrix metallopeptidases (MMP), cyclooxygenase-2 (COX–2) and inducible nitric oxide synthase (iNOS). Thus, induction of the TLR4 pathway is required for LPS-stimulated expression [8]. Heme oxygenase system consists of HO-2 and inducible HO-1 isoforms catalyzes the first and rate-limiting enzymatic step of heme degradation to produce carbon monoxide, iron and biliberdine and is converted to bilirubin [9,10,11]. Among them, HO-1, the inducible isoform of HO, provides a defense mechanism against a series of external stimuli, including cytokines, oxidants, hypoxia and pharmacological agents, exerting macrophage differentiation and major immune regulatory functions [12,13]. Also, HO-1 is upregulated by stress stimuli and is governed by signal transduction and transcriptional regulators, including BTB and CNC homology 1 (BACH1) and nuclear factor erythroid-derived 2-related factor 2 (Nrf2), which act as inhibitors and activators of the HO-1 gene [9,13]. In the process of signaling and transcription factor regulation, BACH1 is a gene transcription inhibitor protein of HO-1, it is known that HO-1 expression by lipopolysaccharide (LPS), a Toll-like receptor 4 (TLR4) activator, is regulated by BACH1 in human macrophages [14]. Thus, BACH1 and Nrf2/HO-1 regulation in LPS-induced inflammatory response may be an important component of inflammatory treatment.

Several marine microorganism-derived natural products have been reported with unique structures and promising pharmacological potential [15,16]. Pseudopterosin A is known as an anti-inflammatory natural product of the sea whip *Pseudopterogorgia elisabethae*, and it has been used as active ingredient of Estee Lauder cosmetics products [17,18]. Particularly, marine bacteria have been known as the producers of diverse polyketides with conjugated double bonds, polyols and macrocyclic rings, for example aureoverticillactam and the marinomycins [19,20,21]. These highly modified lipids or polyketides were also reported with anti-fungal, anti-microbial, anti-infective and anti-cancer activities [22,23,24]. Particularly, microorganisms in the genus of *Bacillus* are known as producers of diverse bioactive natural products including seongsanamide A with anti-allergic activity and turnagainolide B with SHIP1 activating property related to anti-inflammatory drug potential [25,26] In 1989, macrolactins A–F were first found as unique chemical entities with 24-member lactone skeleton containing with conjugated double bonds by Fenical and co-workers [27]. The macrolactins have been reported with their diverse biological activities including antiviral, antitumor, antiangiogenic, intestinal bowel disease protecting and bone-remodeling activities [28,29,30,31,32,33,34,35]. As a part of ongoing research to investigate bioactive natural products from marine-derived microorganisms, the extract of culture broth of *Bacillus* sp. HC001 was found to show a potent cytoprotective effect. A series of chromatography studies on the extract led to the isolation of three natural products including a new compound (**2**, 15-*epi*-dihydromacrolactin F, DMF) and two known analogs (**1**, macroalctin A, MA and **3**, macrolactin F, MF). Therefore, this study reports the effects of three compounds on RAW264.7 and BV2 cells stimulated by LPS.

## 2. Result

### 2.1. Structure Elucidation of DMF, MA and MF

**Figure 1:**
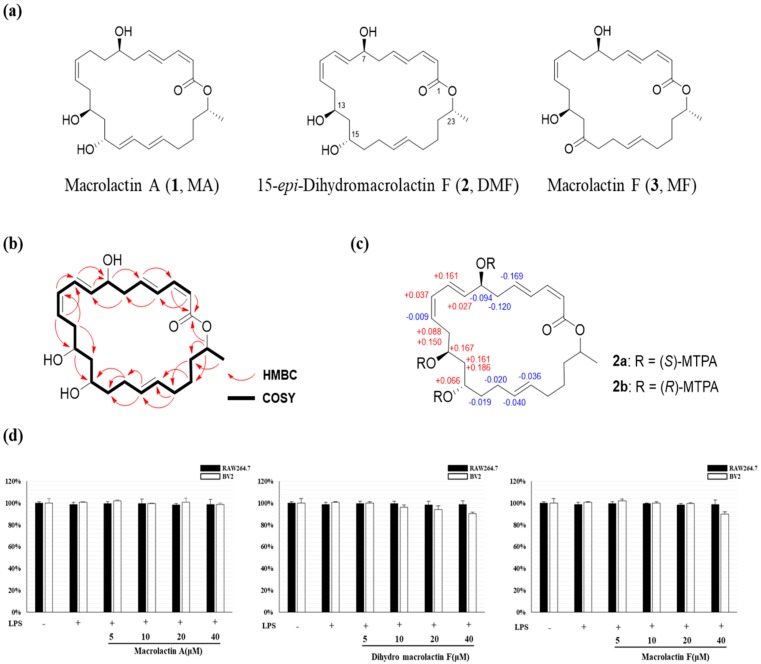
The chemical structures of macrolactin A (**1**, MA), 15-*epi*-dihydromacrolactin F (**2**, DMF) and macrolactin F (**3**, MF) (**a**). Key COSY and HMBC correlations of **2** (**b**). Δδ*_S-R_* values for the configuration analysis of **2a**/**2b** (**c**) and effects of MA, DMF and MF on cell viability on RAW 264.7 and BV2 cells measured by MTT assay after incubation for 24 h (**d**).

Compound **2** was isolated as yellow oil. The molecular formula was determined to be C_24_H_36_O_5_ by a HR-FAB-MS peak at *m*/*z* 405.2641 [M + H]^+^, indicating 7 degrees of unsaturation. The ^1^H and ^13^C NMR spectra indicated the presence of an ester carbonyl group at 166.5 ppm, ten olefinic units (Figure 1b) between 118.2 and 143.3 ppm (Figure S7), four oxygenated methines (δ_H_ 5.00, 4.35, 4.00, 3.98 ppm; δ_C_ 71.2, 71.3, 69.0, 69.5 ppm, respectively), a methyl (δ_H_ 1.25 ppm, δ_C_ 20.2 ppm) and eight methylenes (δ_H_ 2.48, 2.45, 1.97/2.07, 2.04, 1.67, 1.46, 1.44, 1.42 ppm; δ_C_ 41.2, 35.7, 32.4, 29.1, 40.7, 35.2, 36.9, 25.1 ppm, respectively). A series of COSY correlations from H-2 (δ_H_ 5.58 ppm, d, *J* = 11.5 Hz) to H-24 (δ_H_ 1.25 ppm, d, *J* = 6.2 Hz) suggested a long chain type of compound structure. Furthermore, the proton chemical shift of H-23 (δ_H_ 5.00 ppm) was downfield shifted compared to the three other oxygenated methines (H-7, H-13, H-15), suggesting the presence of a cyclic ester linkage between C-1 and C-23 in compound **2**. The geometries of double bonds at C-2, C-4, C-8, C-10 and C-18 were identified as *Z*, *E*, *E*, *Z*, *E* on the basis of their vicinal ^1^H-^1^H coupling constants 11.5, 15.2, 15.2, 11.1 and 15.2 Hz in ^1^H NMR spectrum, respectively. The absolute configuration of C-7, C-13 and C-15 were determined by modified Mosher’s method. The compound 2 was derivatized with (*R*)-(–)- and (*S*)-(+)-ɑ-methoxy-ɑ-(trifluoromethyl)-phenylacetyl chloride (MTPA-Cl) in anhydrous pyridine-*d*_5_ to afford the (*S*)- and (*R*)-MTPA esters (Figures S12–S14). The Δ*δ_S-R_* values of tri-MTPA derivatives of DMF indicated 7*S*, 13*S* and 15*S* configurations. Based on the comparison of chemical shifts and coupling constants of 2 with the reported macrolactins, the configuration of C-23 of **2** was speculated to be *R*. Previously, a 15-epimer of compound **2** was reported as the semisynthetic derivatives of macrolactin F prepared for the structure elucidation of macrolactin B [36]. The absolute configuration of semisynthetic dihydromacrolactin F was revealed to be 7*S*, 13*S*, 15*R*, 23*R*. However, the compound **2** was analyzed to be opposite configuration at C-15 and it was named as 15-*epi*-dihydromacrolactin F (Figure 1a–c).

Compounds **1** and **3** were identified to be macrolactins A and C, respectively, based on the comparison of spectroscopic data including MS spectra as well as ^1^H and ^13^C NMR spectra [27]. 

### 2.2. Effect of MA, DMF and MF on Cell Viability of RAW264.7 and BV2 Cells

First, MTT analysis was performed to evaluate the effect of MA, DMF and MF on cytotoxicity in RAW264.7 and BV2 cells. After treatment with 5 ~ 40 μM MA, DMF and MF for 2 h and then treatment with LPS 1 μg/mL for 24 h, the cell viability of both RAW264.7 and BV2 cells did not affect compared to the control group (Figure 1d). Therefore, subsequent experiments were conducted at concentrations ranging from 5 ~ 40 μM.

### 2.3. Effect of MA, DMF and MF on NO Production and Pro-Inflammatory Cytokines in LPS-Stimulated RAW264.7 and BV2 Cells

**Figure 2:**
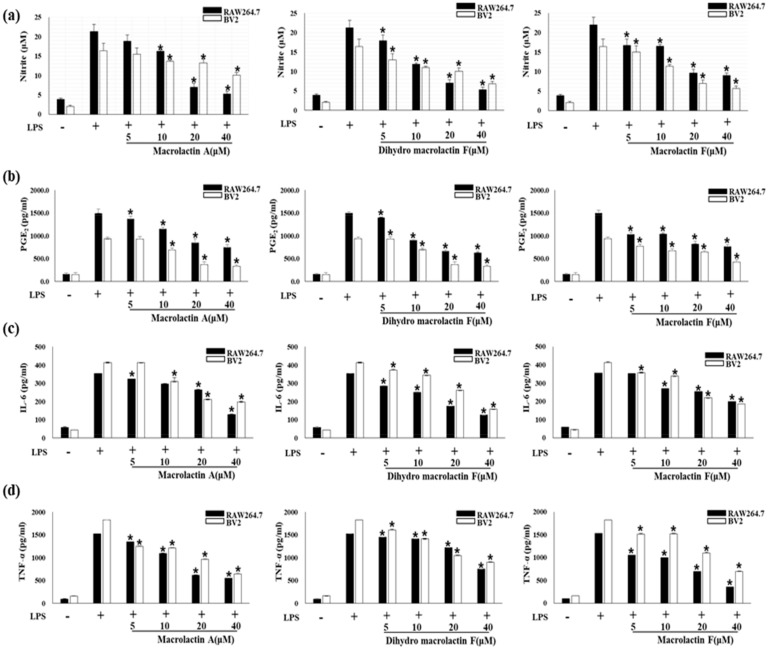
Effect of MA, DMF and MF in LPS-stimulated RAW264.7 and BV2 cells on the production of NO (**a**), PGE2 (**b**), IL-6 (**c**) and TNF-α (**d**). Cells were pretreated for 2 h with indicated concentrations of MA, DMF, MF and stimulated for 24 h with LPS (1 μg/mL). Nitrite concentration, PGE2 assay were performed as described in section materials and methods. Values are expressed as mean ± S.D of three replicates. * *p* < 0.05 compared with LPS-treated group.

Next, pro-inflammatory cytokines are essential mediators for regulating host responses to inflammation. Therefore, we investigated the effects of MA, DMF and MF on NO and PGE_2_ production and pro-inflammatory cytokine expression in LPS-stimulated RAW264.7 and BV2 cells. As a result, MA, DMF and MF down-regulated NO (Figure 2a) and prostaglandin E2 (PGE2) (Figure 2b) production and expression of pro-inflammatory cytokines Interleukin 6 (IL-6) (Figure 2c) and TNF-α (Figure 2d) by LPS stimulation.

### 2.4. Effect of MA, DMF and MF on the Expression of iNOS and COX-2 in LPS-Stimulated RAW264.7 and BV2 Cells

**Figure 3:**
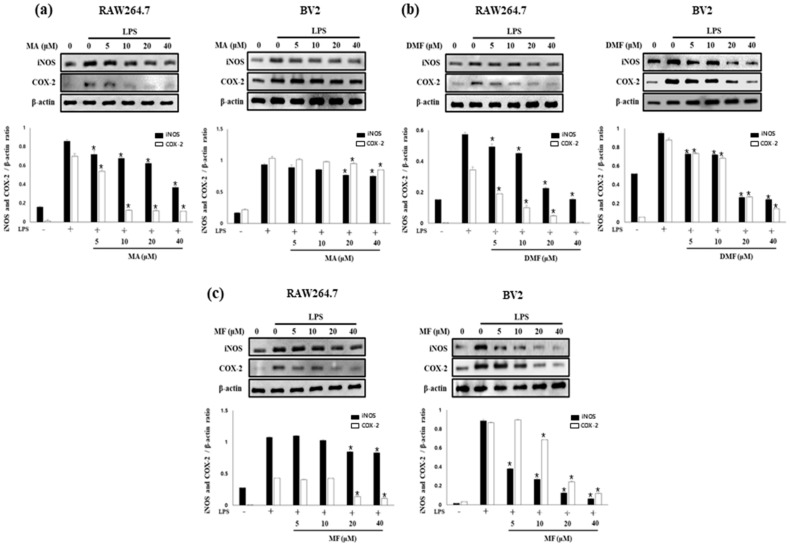
Effects on iNOS and COX-2 protein expression in LPS-induced RAW264.7 and BV2 cells of MA (**a**), DMF (**b**), and MF (**c**). Cells were pretreated for 2 h at various concentrations (5, 10, 20 and 40 μM) and stimulated for 24 h with LPS (1 μg/mL). Values are expressed as mean ± S.D of three replicates. * *p* < 0.05 compared with LPS-treated group.

MA, DMF and MF inhibited NO and PGE2 production in RAW264.7 and BV2 cells. Therefore, the effects of MA, DMF and MF on the expression of iNOS and COX-2 catalyzing the production of NO and PGE2 were evaluated by western blot analysis. As a result it was found that iNOS and COX-2, which were increased in RAW264.7 and BV2 by LPS, were inhibited in a concentration-dependent manner by MA (Figure 3a), DMF (Figure 3b) and MF (Figure 3c). These results suggest that inhibition of iNOS and COX-2 expression by MA, DMF and MF inhibited NO and PGE2 production.

### 2.5. MA, DMF and MF Promote Upregulation of HO-1 Protein Expression and Nucleus Translocation of Nrf2 Protein and Activated Nrf2 Pathway.

**Figure 4:**
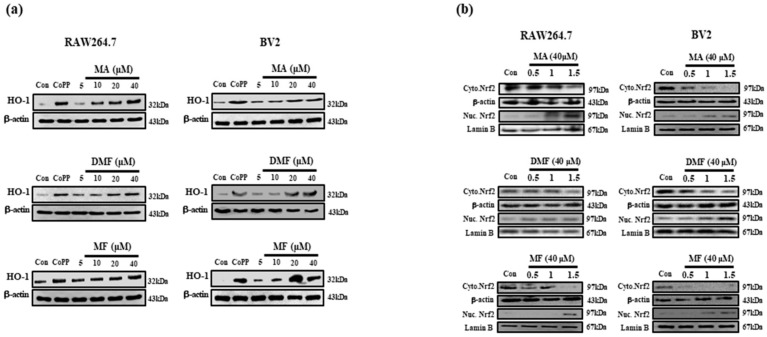
Effects of MA, DMF and MF on HO-1 expression (**a**) and nuclear translocation of Nrf2 (**b**). RAW264.7 and BV2 cells were incubated with indicated concentrations of MA, DMF and MF for 12 h and 40 μM for indicated times.

Previous studies have shown that HO-1 expression mediates anti-inflammatory effects, and the nuclear translocation of activated Nrf2 plays an important role in the major HO-1 induction [37]. Therefore, we identified whether the anti-inflammatory activity of MA, DMF and MF could be associated with HO-1 induction. As a result, MA, DMF and MF increased HO-1 expression in a concentration-dependent manner, and 40 μm expressed HO-1 similar to cobalt protoporphyrin (CoPP), an HO-1 inducer (Figure 4a). In addition, MA, DMF and MF induced Nrf2 nuclear translocation in RAW264.7 and BV2 cells, while Nrf2 levels in nuclear fractions were increased while the cytosolic fraction was degradated (Figure 4b).

### 2.6. MA, DMF and MF Inhibit TLR4 Expression by LPS and Regulate BACH1 and HO-1/Nrf2 Signals.

**Figure 5:**
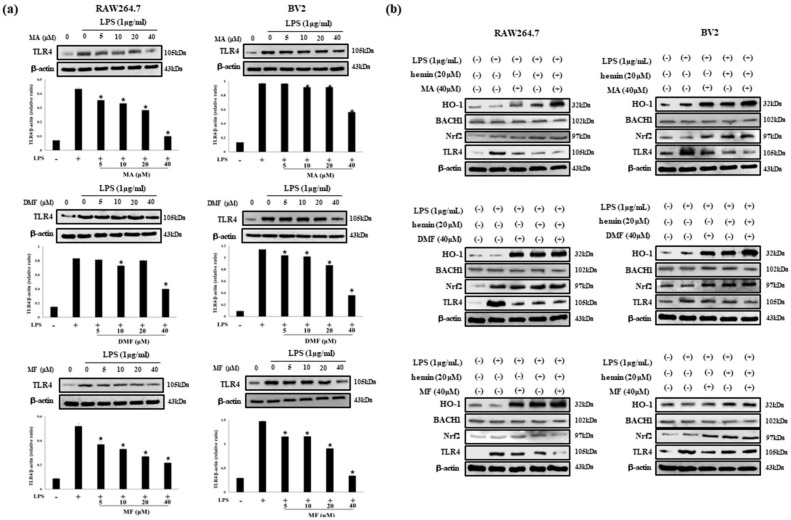
Effect of HO-1 mediates the expression of TLR4 in LPS-stimulated RAW264.7 and BV2 cells (**a**). Cells were incubated with MA, DMF and MF (40 μM) in presence or absence of hemin (20 μM), a HO-1 inducer, and stimulated with or without LPS (1 μg/mL) for 24 h (**b**). Values are expressed as mean ± S.D. of three replicates. * *p* < 0.05 compared with LPS-treated group.

Activation of TLR4 signal transduction by LPS raises pro-inflammatory cytokines. Therefore, we investigated the effect of MA, DMF and MF on TLR4 protein expression by LPS. In addition, the effect of the interaction of Nrf2 and BACH1, a regulator of HO-1, on the expression of TLR4 protein were investigated. First of all, MA, DMF and MF concentration-dependently inhibited TLR4 protein expression induced by LPS (Figure 5a). In addition, BACH1 and TLR4 were down-regulated and Nrf2 was up-regulated according to the expression of HO-1 increased by MA, DMF and MF. These results were confirmed more clearly when the co-treated of HO-1 inducer CoPP and MA, DMF and MF (Figure 5b).

### 2.7. Effect of MA, DMF and MF on LPS-Induced Phosphorylation of TAK1, MAPK and NF-κB

**Figure 6:**
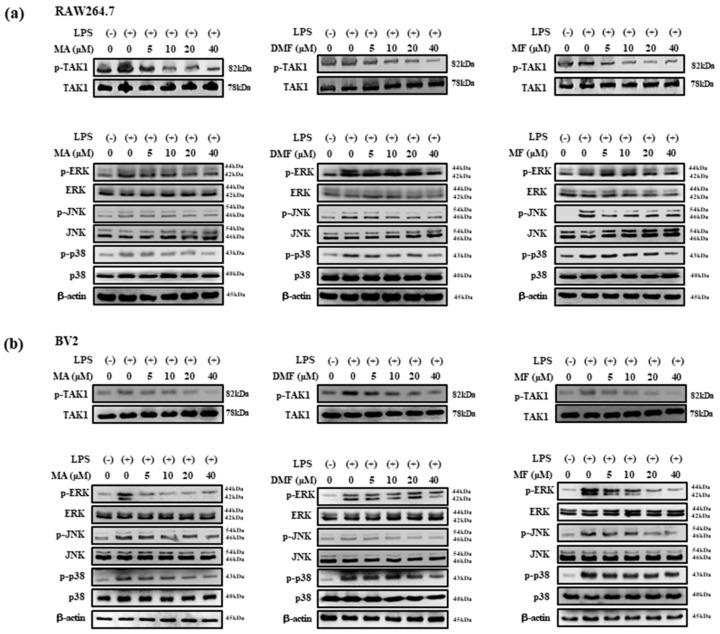
The effect of MA, DMF and MF on the activation of MAPK pathways in LPS-stimulated: RAW264.7 cells (**a**) and BV2 cells (**b**). The cells were incubated for 24 h, the next cells were pretreated with indicated concentration of MA, DMF and MF for 2 h, and stimulated with LPS for 30 min. The total proteins of the cells were prepared and the expressions of phosphorylated JNK, ERK and p38 were detected by western blot.

**Figure 7:**
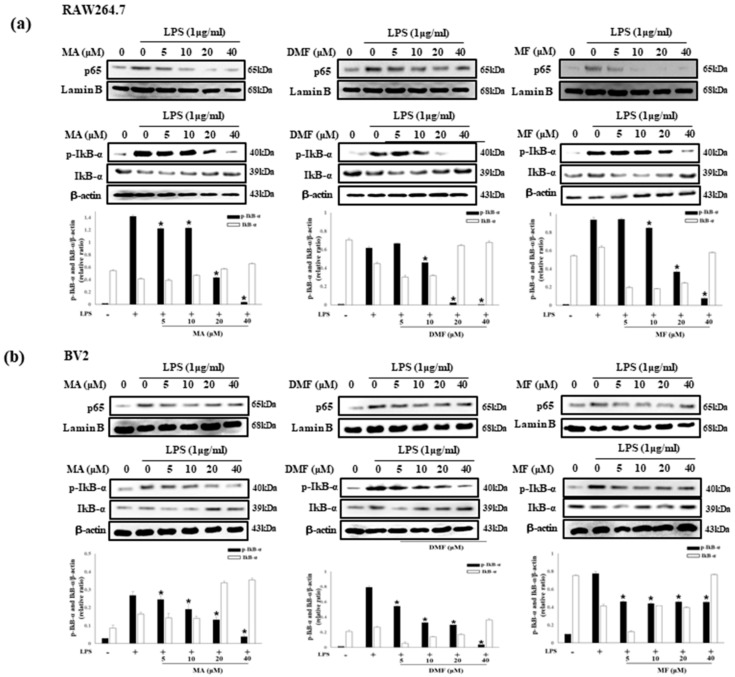
Effect of MA, DMF and MF on NF-κB and activation in LPS-stimulated RAW 264.7 (**a**) and BV2 cells (**b**). Cells were pre-incubated with or without MA, DMF and MF for 2 h, following treatment with LPS for 30 min. Relative protein expressions of cytosolic and nuclear p65, IkBα and p-IkBα to those in control were subjected to western blot analysis. * *p* < 0.05 compared with LPS-treated group.

TAK1 is an upstream signaling molecule of NF-κB that regulates inflammatory genes and pro-inflammatory cytokines, and MAPK is known to be involved in the regulation of pro-inflammatory mediator expression in LPS-treated RAW 264.7 cells [38]. Therefore, we investigated the effect of MA, DMF and MF on the phosphorylation of TAK1, MAPK and NF-κB in LPS-stimulated RAW264.7 and BV2 cells through western blot analysis. MA, DMF and MF inhibited the expression of phosphorylated TAK1 (Figure 6a), ERK, JNK and p38 (Figure 6b), and also inhibited the expression of NF-κB p65 and phosphorylated IκBα (Figure 7a,b).

## 3. Discussion

The main pattern recognition receptor TLR4 in LPS-induced inflammation is involved in the expression of pro-inflammatory genes and secretion of pro-inflammatory molecules by NF-κB and MAPK activation, which plays an important role in the release of pro-inflammatory mediators such as TNF-α, IL-1β, IL-6 and PGE2 [39,40]. In this study, MA, DMF and MF inhibited the expression and production of pro-inflammatory cytokines and pro-inflammatory mediators in LPS-stimulated RAW264.7 and BV2 cells and inhibited TLR4 protein expression by LPS-stimulated expression. LPS, a major component of *E. coli* cell membranes, is widely used in in vivo and in vitro inflammation models, and studies previously reported that activation of MAPK and NF-κB by LPS regulates many genes in the inflammatory response [41]. In our results, MA, DMF and MF inhibited the phosphorylation of MAPK and NF-κB in inflammatory responses induced by LPS. Therefore, it is suggested that the inhibitory effect of the expression of pro-inflammatory cytokines and pro-inflammatory mediators are shown by inhibiting the activities of MAPK and NF-κB.

Inflammation is a host response to various stimuli, including oxidative stress and infection leading to the release of large amounts of inflammatory mediators, and in this pathophysiological state HO-1 is a potent antioxidant that is activated and exerts a cellular protective effect [42,43]. Nuclear transcription factor Nrf2 is known to be one of the important antioxidant regulators involved in maintaining the redox state for defense against oxidative stress in cells, and this defense mechanism is mediated transcription and protein of ARE- and Nrf2 dependent antioxidant factors including HO-1 and Gpx1 [44,45]. BACH1 is a heme sensing protein that loses its inhibitory activity upon heme levels and heme binding in cells, and high levels of heme induce degradation of BACH1 [46]. In particular, BACH1 and Nrf2 act as inhibitors and activators of the HO-1 gene, and previously reported that, during the signaling of TLR4 by LPS, the HO-1 expression is regulated by BACH1 and activates the Nrf2 antioxidant stress pathway [37]. Therefore, we investigated the correlation between BACH1 and Nrf2/HO-1 in RAW264.7 and BV2 cells activated with TLR4 signaling by LPS stimulation. As a result, MA, DMF and MF increased HO-1 expression in a concentration-dependent manner, and inhibited TLR4 activation by LPS. In addition, the increased HO-1 expression, according to the regulation of Nrf2 was shown to decrease the expression of BACH1, and this result became more apparent when CoPP (HO-1 inducer) is processed simultaneously. Nrf2/HO-1 signals have been shown to inhibit inflammation by modulating various inflammatory signals, including MAPK, STAT3 and AKT, among them activation of MAPK is induced by TAK1, and expression of TAK1 enhances expression of Nrf2 by activating antioxidant response element (ARE) [47]. TLR4 mediated intracellular signaling pathways in these inflammatory responses are important for induction of innate immune responses [7]. In particular, TLR4-mediated signaling pathways have been shown to inhibit the transcription of cytokines and chemokines that participate in the initiation or regulation of the inflammatory response by inhibiting activation of NF-κB and MAPK [48]. 

## 4. Summary and Conclusion

To summarize this study, the effects of microorganism-derived chemical MA, DMF and MF on LPS-stimulated RAW264.7 and BV2 cells were investigated. In the study, MA, DMF and MF influenced LPS-induced TLR4 activity and inhibition by regulating BACH1 and HO-1/Nrf2 signals. In addition, MA, DMF and MF down-regulated pro-inflammatory cytokines and mediators by inhibiting the phosphorylation of MAPK and NF-κB p65. Thus, this study suggests that marine microbial chemicals MA, DMF and MF are potential therapeutic agents for inflammatory diseases.

## 5. Materials and Methods

### 5.1. General Experimental Procedures

Optical rotations were measured on DIP-1000 digital polarimeter (JASCO Corporation, Tokyo, Japan). UV data were determined with a JASCO UV/VIS-Spectrophotometer (JASCO Corporation, Tokyo, Japan). ^1^H and ^13^C NMR data were obtained from Bruker AVANCE 250 spectrometer (Bruker BioSpin Corporation, Billerica, MA, USA) and Jeol ECA-500 spectrometer (Jeol Ltd., Akishima, Tokyo, Japan) using TMS as internal standard. LR-ESI-MS were recorded on Agilent 6120 quadrupole MSD consisting of 1260 Infinity pump, 1260 Infinity autosampler, 1260 Infinity DAD detector and an Openlab ChemStation for data acquisition and processing. HR-FAB-MS spectra were performed on JEOL JMS-600W high-resolution mass spectrometer. Preparative HPLC was achieved with Gilson TRILUTION LC system with 321 pump, and UV/Vis–155 detector using Phenomenex Luna (Phenomenex, Torrance, CA, USA) C18 column (250 × 21 mm, 20 μm).

### 5.2. Isolation of Bacterial Strain and Taxonomic Identification

The microbial strain (HC001) was isolated from the marine sediment collected from Je-ju Island in 2013. The dried sediment was stamped on SYP SW solid medium (soluble starch 10 g/L; yeast extract 4 g/L; peptone 2 g/L; agar 16 g/L; sterilized and filtered sea water 1 L) plate for several days at 27 °C, and the observed colonies were isolated. One of the isolated strains was identified as *Bacillus* sp. HC001 based on 16S rDNA sequence (blast top strain: *Bacillus amyloliquefaciens* strain Lx–11, 99.79% similarity) and the strain was stored at College of Pharmacy, Yeungnam University.

### 5.3. Culture of Bacterial Strain

The strain *Bacillus* sp. HC001 was inoculated in SYP SW medium (soluble starch 10 g/L; yeast extract 4 g/L; peptone 2 g/L; sterilized and filtered sea water 1 L) as seed culture. The seed cultures were incubated in a shaking incubator with 150 rpm at 25 °C for 3 days. The seed cultures were inoculated in SYP medium (1 L of media in 2.5 L culture flask, totally 35 flasks) and incubated in a shaking incubator with 150 rpm at 25 °C for 7 days.

### 5.4. Extraction and Isolation

The culture broth of *Bacillus* sp. HC001 was extracted twice with the same volume of ethyl acetate (EtOAc) and the combined organic layer was concentrated *in vacuo* to give 1.9 g of crude extract. The crude extract was subjected to vacuum liquid chromatography (VLC) using step gradient elution with 0–100% MC in MeOH to obtain fractions A–G. fraction E (195.5 mg) was further separated using reverse-phase preparative HPLC (gradient elution, a mixture of 50% acetonitrile changed to 60% acetonitrile in water for 1 h, flow rate 6 mL/min) to afford macrolactin A (**1**, MA, 9.7 mg, Rt = 29.2 min), 15-*epi*-dihydromacrolactin F (**2**, DMF, 4.7 mg, Rt = 36.6 min) macrolactin F (**3**, MF, 3.2 mg, Rt = 44.0 min) were obtain.

15-*epi*-Dihydromacrolactin F (**2**): yellow oil, [α${]}_{D}^{25}$ −19 (c 0.1, MeOH); UV (MeOH) λ_max_ (log ε) 235 (4.37) and 261 (4.27) nm; ^1^H NMR (500 MHz, CDCl_3_) *δ* 7.26 (dd, 15.2, 11.5, H-4), 6.57 (dd, 15.2, 11.1, H-9), 6.55 (dd, 11.5, 11.2, H-3), 6.13 (dd, 11.1, 10.8, H-10), 6.06 (td,15.2, 7.7, H-5), 5.76 (dd, 15.2, 5.9,H-8), 5.58 (d, 11.5, H-2), 5.50 (td, 10.8, 8.3, H-11), 5.42 (m, H-19), 5.41 (m, H-18), 5.00 (m, H-23), 4.35 (td, 6.3, 5.9, H-7), 4.00 (m, H-15), 3.98 (m, H-13), 2.48 (m, H-6), 2.45 (m, H-12), 2.07 (m, H-20a), 2.04 (m, H-17), 1.97 (m, H-20b), 1.67 (m, H-14), 1.65 (m, H-22a), 1.46 (m, H-22b), 1.44 (m, H-16), 1.42, (m, H-21), 1.25 (d, 6.2, H-24); ^13^C NMR (125 MHz, CDCl_3_) *δ* 166.5 (C-1), 143.3 (C-3), 139.9 (C-5), 135.8 (C-8), 130.9 (C-19), 130.5 (C-10), 130.4 (C-18), 129.9 (C-4), 128.3 (C-11), 125.6 (C-9), 118.2 (C-2), 71.3 (C-7), 71.2 (C-23), 69.5 (C-13), 69.0 (C-15), 41.2 (C-6), 40.7 (C-14), 36.9 (C-16), 35.7 (C-12), 35.2 (C-22), 32.4 (C-20), 29.1 (C-17), 25.1 (C-21), 20.2 (C-24); HRFABMS *m*/*z* 405.2641 [M + H]^+^ (calcd for C_24_H_37_O_5_^+^, 405.2636).

### 5.5. Preparation of (R/S)-tris-MTPA Esters of Compound **2**

Compound **2** (1 mg) was dissolved in 0.5 mL of prydine-*d*_5_ into 4 mL vial, and then 2 mg of 4-dimethylaminopyridine (DMAP) was added into the vial at room temperature for 5 min. To the reaction vial, (*R*)-(−)-ɑ-methoxy-ɑ-(trifluoromethyl)-phenylacetyl chloride (*R*-MTPA-Cl) was added and the mixture was transferred into a 5 mm dried NMR tube at RT for 16 h with the monitoring by ^1^H NMR experiment. The reaction mixture was transferred to vial, concentrated under N_2_ gas and extracted with *n*-hexane. Tris-(*S*)-MTPA ester of **2** (**2a**, 0.4 mg) was purified by analytical HPLC using Inertsil HPLC column (SIL 100A 5 μm, 4.6 × 250 mm, *n*-hexane: 10% isopropyl alcohol in *n*-hexane = 99: 1, flow rate 1 mL/min).

Tris-(*R*)-MTPA ester of **2** (**2b**) was obtained by the entirely analogous method described above, using (*S*)-(+)-ɑ-methoxy-ɑ-(trifluoromethyl)-phenylacetyl chloride (*S*-MTPA-Cl). The purified **2b** (0.5 mg) was obtained by the same method used in the isolation of **2a**.

### 5.6. Materials and Reagents

Dulbecco’s modified Eagle’s medium (DMEM) and fetal bovine serum (FBS) was purchased from Welgene (Korea). Penicillin–streptomycin was obtained from Gibco (Grand Island, NY, USA). LPS (*Escherichia coli*) were obtained from Invivogen (San Diego, CA, USA). Also, Primary antibodies were purchased from Cell Signaling Tech, Santa Cruz Biotech and Enzo Life Sciences (p38 cat#9212S, p-p38 cat#9211, p-JNK cat#9251, JNK cat#9252, ERK cat#9102, p-ERK cat#9101, β-actin cat#4967, Nrf2 cat#12721, p-TAK1 cat#4531, TAK1 cat#5206, lamin B cat#12586, NF-κB p65 cat#8242, IκBα cat#9242 and p-IκBα cat#9246, Cell Signaling Tech, MA, USA; BACH1 cat#sc-271211, TX, USA; and HO-1 cat#ADI-SPA-895-F, Enzo Life Sciences, USA). Secondary antibodies were purchased from Santa Cruz Biotechnology (Santa Cruz, CA, USA) and used.

### 5.7. Cell Culture

RAW264.7 macrophages and BV2 microglia were purchased from American Type Culture Collection (ATCC). RAW264.7 and BV2 cells were cultured in DMEM medium (supplemented with 10% heat-inactivated FBS, penicillin (100 units/mL), streptomycin (100 mg/mL) and L-glutamine (2 mM)) and were grown at 37 °C in a humidified atmosphere containing 5% CO_2_

### 5.8. Cell Viability Assay

RAW264.7 and BV2 cells were maintained at 1 × 10^5^ cells/mL in 96-well plates at Dulbecco’s modified eagle medium (DMEM) supplemented with 10% heat-inactivated fetal bovine serum (FBS), penicillin (100 U/mL), streptomycin (100 mg/L), L-glutamine (2 mM) incubated at 37 °C in a humidified atmosphere containing 5% CO_2_ and treated with LPS (1 μg/mL) and various concentrations of Macrolactin A (MA), Dihydro macrolactin F (DMF) and Macrolactin F (MF) for 24 h. [4,5-dimethylthiazol-2-yl]-2,5-diphenyltetrazolium bromide (MTT) 100 mg/mL adding after incubating for 2 h. The formazan formed was dissolved in DMSO, and the optical density was measured at 540 nm.

### 5.9. NO Production and Assays

RAW264.7 and BV2 cells (1 × 10^6^ cells/mL) in 96-well plates and incubated overnight. Samples of various concentrations were treated for 2 h, followed by LPS (1 μg/mL) for 24 h. The absorbance of the final product was then measured spectrophotometrically at 540 nm using an ELISA plate reader according to the depicted method. Nitrite concentration in the measured sample was calculated from the calibration curve.

### 5.10. Cytokine Analysis

RAW264.7 and BV2 cells cultured in 24-well plates (6 × 10^5^ cells/well) were treated with MA, DMF, MF and LPS (1 μg/mL) for 24 h. The supernatant of each well was harvested; the PGE2 content was measured using the mouse PGE2 ELISA kit (R & D Systems) and the IL-6 and TNF-α contents were determined using the mouse IL-6 ELISA kit (R & D Systems) and mouse TNF-α ELISA kit (Enzo Life Sciences) according to the manufacturers protocol. The microplate reader was measured at a wavelength of 540 nm.

### 5.11. Western Blot Analysis

RAW264.7 and BV2 cells were treated with 5, 10, 20 and 40 μM of MA, DMF and MF for 2 h subsequent to the addition of 1 μg/mL of LPS. The protein samples were prepared using RIPA lysis buffer (Thermo Scientific, MA, USA), which contained protease and phosphatase inhibitors, and quantified using a BCA protein estimation kit (Thermo Scientific, MA, USA). A total of 30 μg of protein was loaded on 12% SDS PAGE and transferred onto a nitrocellulose (NC) membrane (Millipore, MA, USA). Then, the membrane was blocked with 5% skimmed milk in PBST (PBS containing 0.05% Tween-20) for 2 h, and incubated with the desired primary antibody overnight at 4 °C. Several groups of samples were separated by SDS–PAGE for western blot analysis and then incubated with primary antibody overnight at 4 °C. The secondary antibody was then incubated for 2 h at room temperature. Immune blotting reagents ECL-plus (GE Healthcare Life Science, Tokyo, Japan) were then used to detect immunoreactive bands using FujiFilm LAS4000. Gray values of the bands were quantified by J-Image software and quantified by β-actin.

### 5.12. Cytosolic and Nuclear Protein Extraction

RAW264.7 macrophages and BV2 microglia cells were seeded at 5 × 10^6^ cells/mL. The harvested cells were then lysed on ice for 20 min with radioimmunoprecipitation assay (RIPA) buffer (Thermo Fisher Scientific, MA, Waltham, MA), and the isolated cytoplasm and nuclei were removed using the NE-PER nuclear and cytoplasmic extraction reagent kit (Pierce Biotechnology, Rockford, IL, USA) according to the manufacturers instructions. Each was used for western blot analysis.

### 5.13. Statistical Analysis

All experiments were presented as mean ± SD from at least 3 independent experiments, all statistical analysis was performed with SPSS 12.0.1 for Windows, using one-way analysis of variance (ANOVA). The p-value was considered statistically significant when it was less than 0.05.

## Supplementary Materials

The following are available online at https://www.mdpi.com/1420-3049/25/3/656/s1. NMR spectra of **1**–**3** and the Mosher’s esters (**2a** and **2b**) along with HR-FAB-MS of **2**., **Figure S1:** 1H NMR (250 MHz, CDCl_3_) spectrum of macrolactin A (**1**), **Figure S2:** 13C NMR (62.5 MHz, CDCl_3_) spectrum of macrolactin A (**1**), **Figure S3:** 1H-1H COSY (250 MHz, CDCl_3_) spectrum of macrolactin A (**1**), **Figure S4:** HMQC (250 MHz, CDCl_3_) spectrum of macrolactin A (**1**), **Figure S5:** HMBC (250 MHz, CDCl_3_) spectrum of macrolactin A (**1**), **Figure S6:** 1H NMR (500 MHz, CDCl_3_) spectrum of 15-*epi*-dihydromacrolactin F (**2**), **Figure S7:** 13C NMR (125 MHz, CDCl_3_) spectrum of 15-*epi*-dihydromacrolactin F (**2**), **Figure S8:** 1H-1H COSY (500 MHz, CDCl_3_) spectrum of 15-*epi*-dihydromacrolactin F (**2**), **Figure S9:** HMQC (500 MHz, CDCl_3_) spectrum of 15-*epi*-dihydromacrolactin F (**2**), **Figure S10:** HMBC (500 MHz, CDCl_3_) spectrum of 15-*epi*-dihydromacrolactin F (**2**), **Figure S11:** HR- FAB-MS of 15-*epi*-dihydromacrolactin F (**2**), **Figure S12:** 1H NMR (500 MHz, CDCl_3_) spectrum of *tris*-(*S*)-MTPA *e*ster of **2** (**2a**), **Figure S13:** 1H-1H COSY (500 MHz, CDCl_3_) spectrum of *tris*-(*S*)-MTPA *e*ster of **2** (**2a**), **Figure S14:** 1H NMR (500 MHz, CDCl_3_) spectrum of *tris*-(*R*)-MTPA *e*ster of **2** (**2b**), **Figure S15:** 1H-1H COSY (500 MHz, CDCl_3_) spectrum of *tris*-(*R*)-MTPA *e*ster of **2** (**2b**), **Figure S16:** 1H NMR (500 MHz, CD_3_OD) spectrum of macrolactin F (**3**), **Figure S17:** 13C NMR (125 MHz, CD_3_OD) spectrum of macrolactin F (**3**), **Figure S18:** 1H-1H COSY (500 MHz, CD_3_OD) spectrum of macrolactin F (**3**), **Figure S19:** HMQC (500 MHz, CD_3_OD) spectrum of macrolactin F (**3**), **Figure S20:** HMBC (500 MHz, CD_3_OD) spectrum of macrolactin F.(**3**).
