# Supplementary material for: Marine Microorganism-Derived Macrolactins Inhibit Inflammatory Mediator Effects in LPS-Induced Macrophage and Microglial Cells by Regulating BACH1 and HO-1/Nrf2 Signals through Inhibition of TLR4 Activation
Source: Molecules. 2020 Feb 4;25(3):656. doi: 10.3390/molecules25030656 (PMC7037854; doi:10.3390/molecules25030656)
Supplement: Supplementary file 1 [file molecules-25-00656-s001.pdf]

molecules

Supplementary Materials

Marine microorganism-derived macrolactins inhibit inflammatory mediator effects in LPS-induced macrophage and microglial cells by regulating BACH1 and HO-1/Nrf2 signals through inhibition of TLR4 activation

Eun-Nam Kim[1#], Ming Gao[2#], Hyukjae Choi[2*] and Gil-Saeng Jeong[1*]

[1]College of Pharmacy, Keimyung University, 1095 Dalgubeol-daero, Daegu 42601, Republic of Korea; enkimpharm@gmail.com (E-N. K.); gsjeong@kmu.ac.kr (G-S. K.)
[2]College of Pharmacy, Yeungnam University, 280 Daehak-ro, Gyeongsan 38541, Republic of Korea; gaoming104@hotmail.com (M. G.); h5choi@yu.ac.kr (H. C.)
*Correspondence to: Prof. Gil-Saeng Jeong and Prof. Hyukjae Choi
[#]These two authors contributed equally to this work.

List of Figures

Figure S1: ^{1}H NMR (250 MHz, $CDCl_3$) spectrum of macrolactin A (**1**)
Figure S2: ^{13}C NMR (62.5 MHz, $CDCl_3$) spectrum of macrolactin A (**1**)
Figure S3: ^{1}H-^{1}H COSY (250 MHz, $CDCl_3$) spectrum of macrolactin A (**1**)
Figure S4: HMQC (250 MHz, $CDCl_3$) spectrum of macrolactin A (**1**)
Figure S5: HMBC (250 MHz, $CDCl_3$) spectrum of macrolactin A (**1**)
Figure S6: ^{1}H NMR (500 MHz, $CDCl_3$) spectrum of 15-*epi*-dihydromacrolactin F (**2**)
Figure S7: ^{13}C NMR (125 MHz, $CDCl_3$) spectrum of 15-*epi*-dihydromacrolactin F (**2**)
Figure S8: ^{1}H-^{1}H COSY (500 MHz, $CDCl_3$) spectrum of 15-*epi*-dihydromacrolactin F (**2**)
Figure S9 : HMQC (500 MHz, $CDCl_3$) spectrum of 15-*epi*-dihydromacrolactin F (**2**)
Figure S10 : HMBC (500 MHz, $CDCl_3$) spectrum of 15-*epi*-dihydromacrolactin F (**2**)
Figure S11: HR- FAB-MS of 15-*epi*-dihydromacrolactin F (**2**)
Figure S12: ^{1}H NMR (500 MHz, $CDCl_3$) spectrum of *tris*-(*S*)-MTPA *e*ster of **2** (**2a**)
Figure S13: ^{1}H-^{1}H COSY (500 MHz, $CDCl_3$) spectrum of *tris*-(*S*)-MTPA *e*ster of **2** (**2a**)
Figure S14: ^{1}H NMR (500 MHz, $CDCl_3$) spectrum of *tris*-(*R*)-MTPA *e*ster of **2** (**2b**)
Figure S15: ^{1}H-^{1}H COSY (500 MHz, $CDCl_3$) spectrum of *tris*-(*R*)-MTPA *e*ster of **2** (**2b**)
Figure S16: ^{1}H NMR (500 MHz, CD_3OD) spectrum of macrolactin F (**3**)
Figure S17: ^{13}C NMR (125 MHz, CD_3OD) spectrum of macrolactin F (**3**)
Figure S18: ^{1}H-^{1}H COSY (500 MHz, CD_3OD) spectrum of macrolactin F (**3**)
Figure S19: HMQC (500 MHz, CD_3OD) spectrum of macrolactin F (**3**)
Figure S20: HMBC (500 MHz, CD_3OD) spectrum of macrolactin F (**3**)

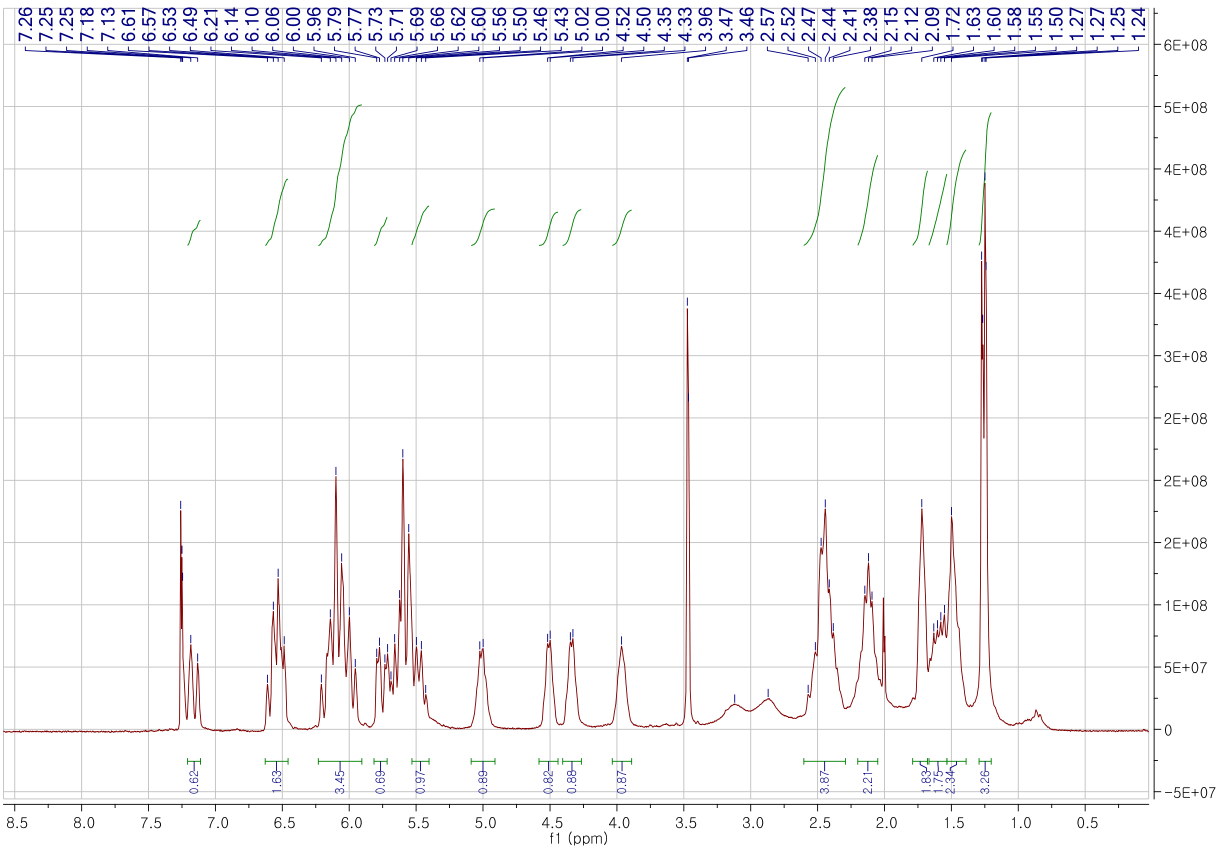

Figure S1: ^{1}H NMR (250 MHz, $CDCl_3$) spectrum of macrolactin A (**1**)

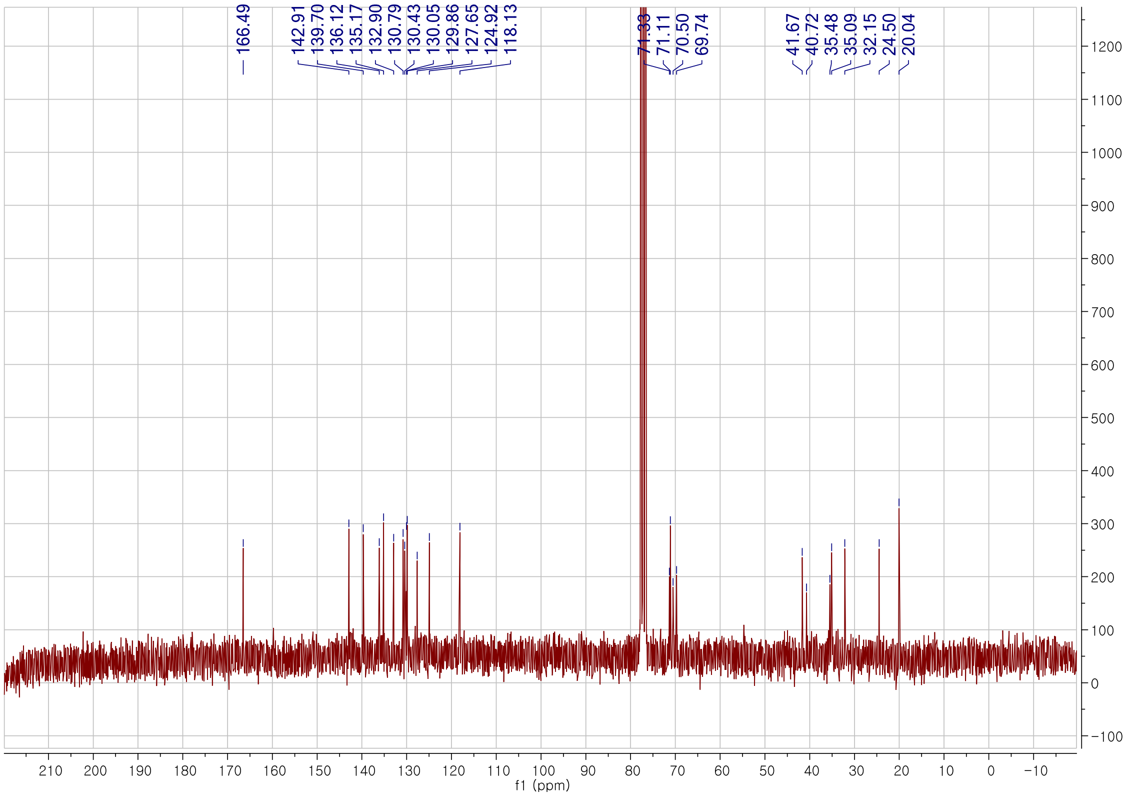

Figure S2. ^{13}C NMR (62.5 MHz, $CDCl_3$) spectrum of macrolactin A (**1**)

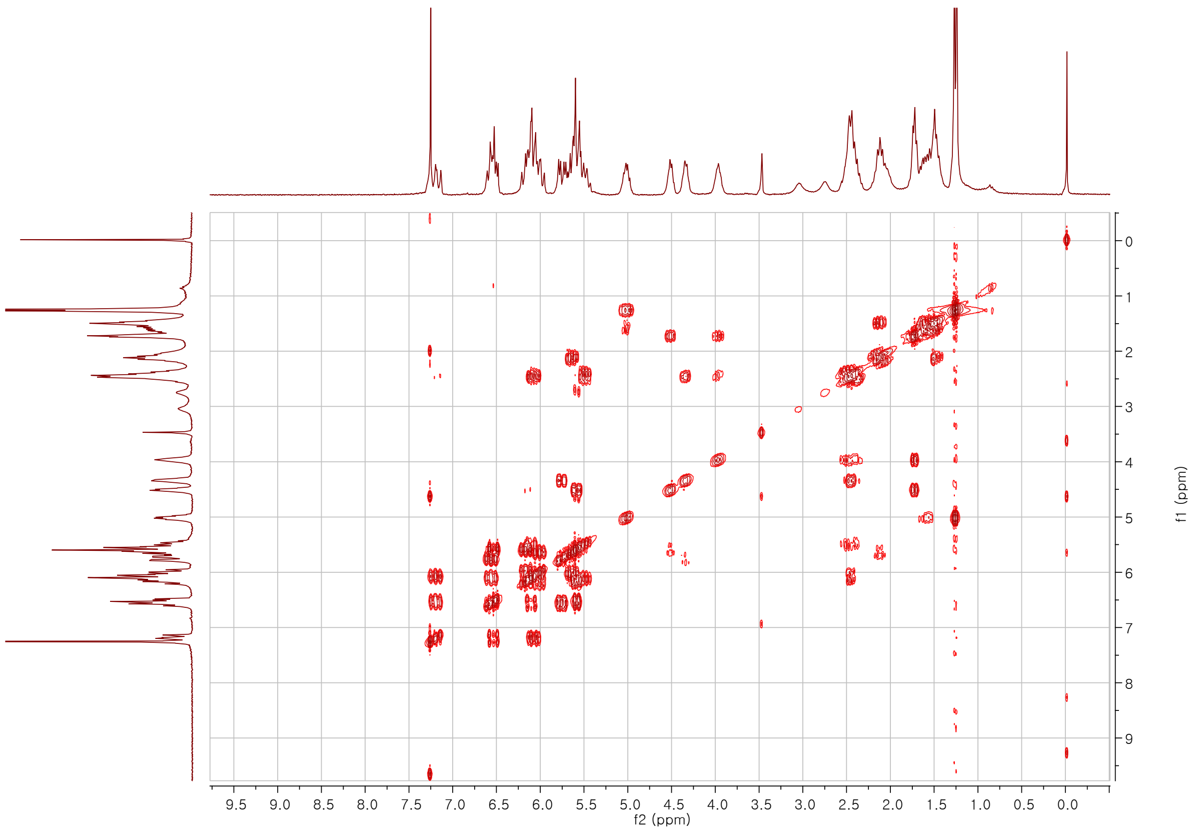

Figure S3. ^{1}H-^{1}H COSY (250 MHz, $CDCl_3$) spectrum of macrolactin A (**1**)

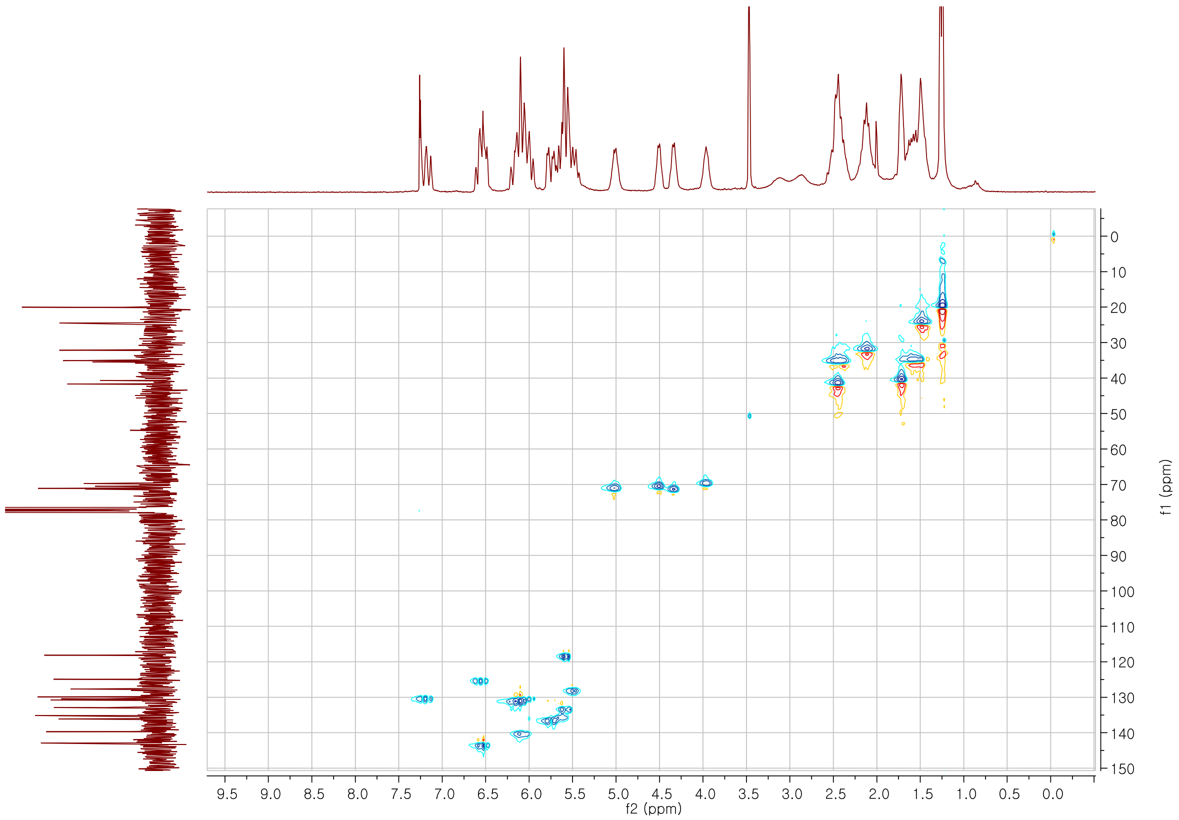

Figure S4. HMQC (250 MHz, $CDCl_3$) spectrum of macrolactin A (**1**)

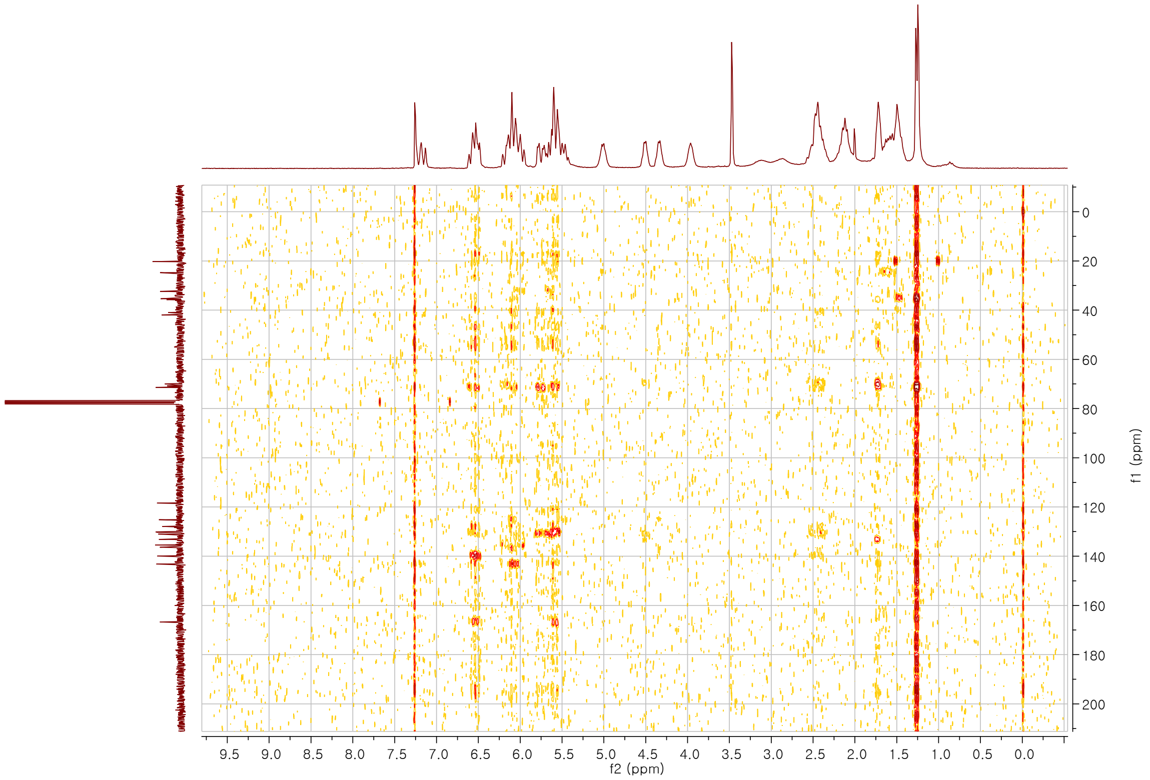

Figure S5. HMBC (250 MHz, $CDCl_3$) spectrum of macrolactin A (**1**)

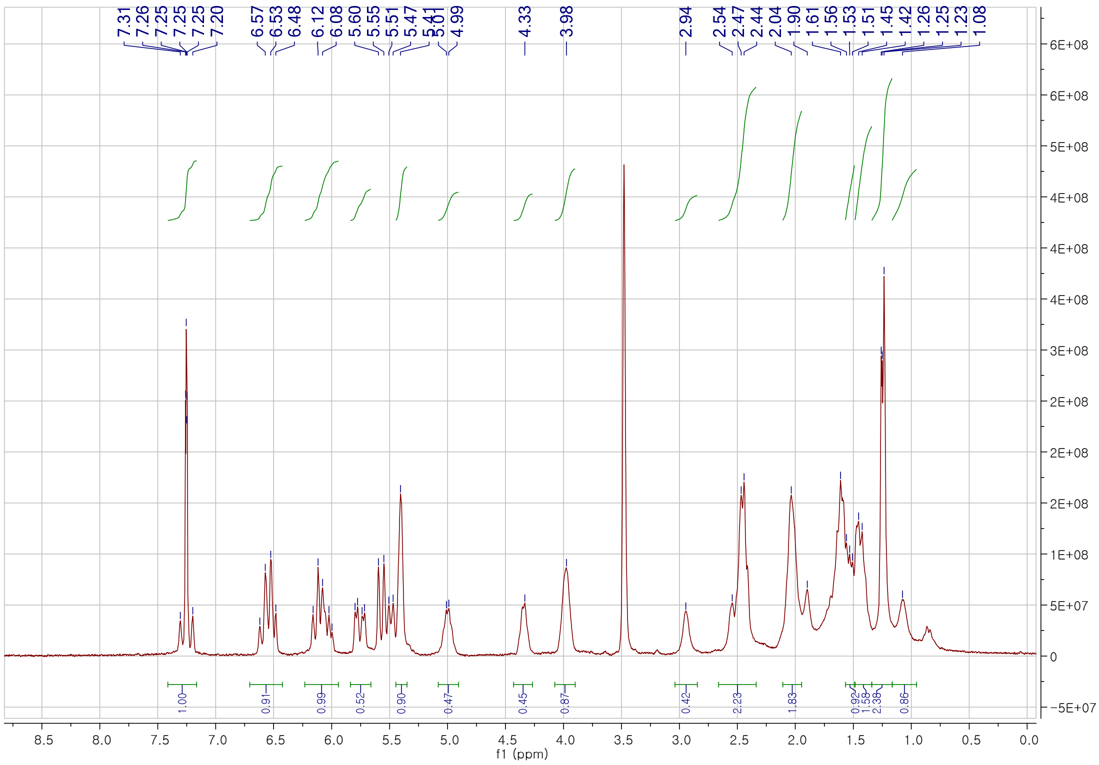

Figure S16. ^{1}H NMR (500 MHz, $CDCl_3$) spectrum of 15-*epi*-dihydromacrolactin F (**2**)

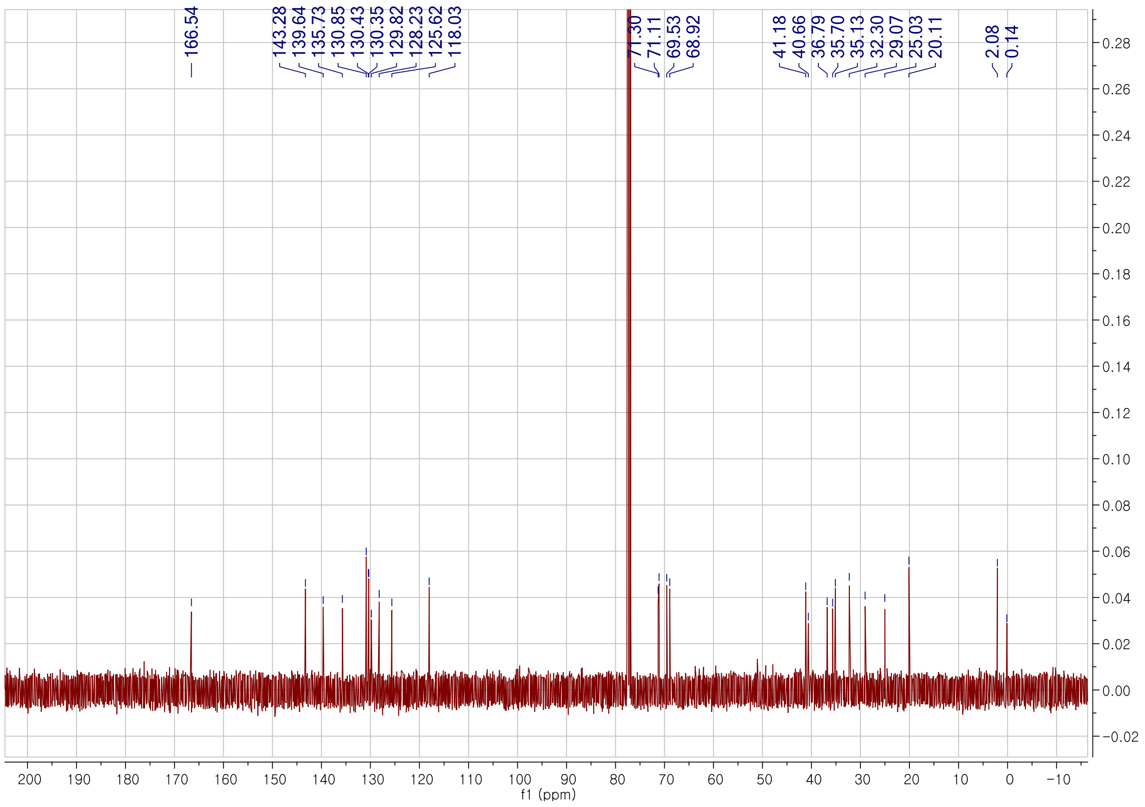

Figure S7. ^{13}C NMR (125 MHz, $CDCl_3$) spectrum of 15-*epi*-dihydromacrolactin F (**2**)

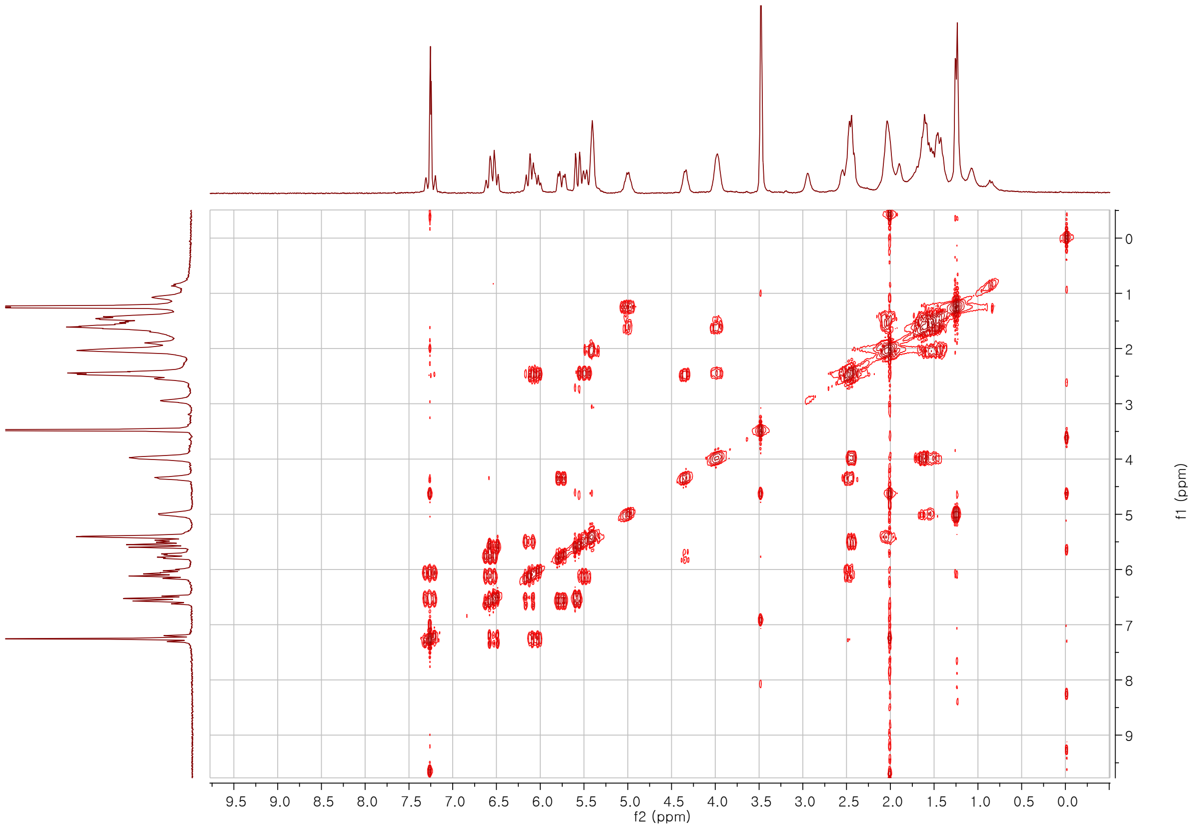

Figure S8. ^{1}H-^{1}H COSY (500 MHz, $CDCl_3$) spectrum of 15-*epi*-dihydromacrolactin F (**2**)

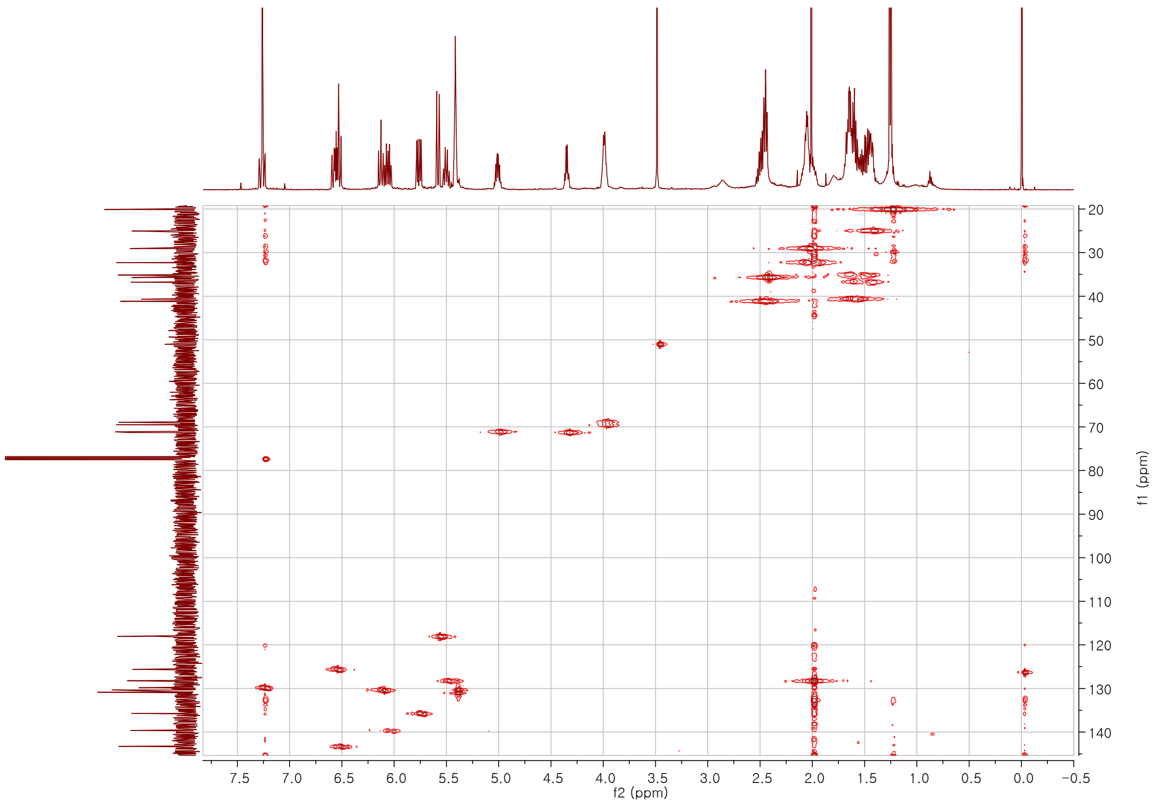

Figure S9. HMQC (500 MHz, $CDCl_3$) spectrum of 15-*epi*-dihydromacrolactin F (**2**)

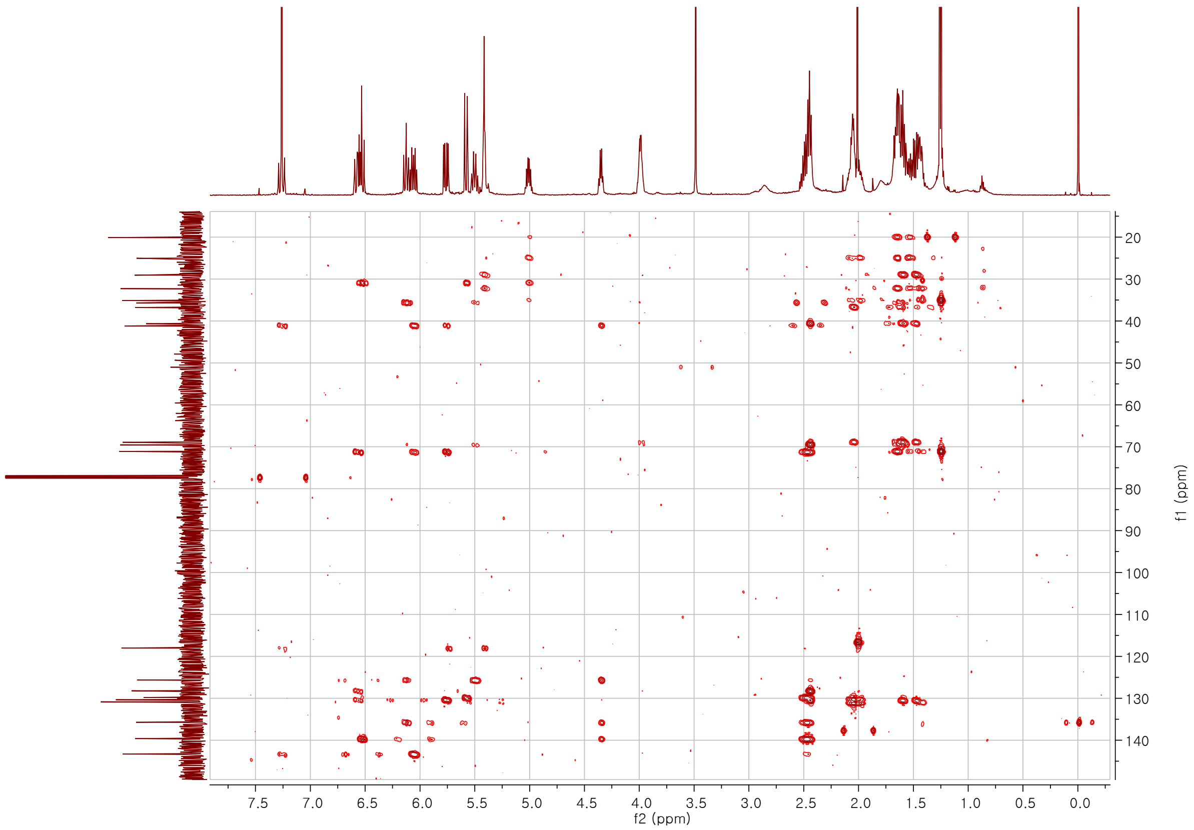

Figure S10. HMBC (500 MHz, $CDCl_3$) spectrum of 15-*epi*-dihydromacrolactin F (**2**)

[Mass Spectrum]
Data : FAB-M156 Date : 18-Jun-2015 16:04
Sample: (S)Tri-mosher ester HC001 E4
Note : m-NBA
Inlet : Direct Ion Mode : FAB+
Spectrum Type : Normal Ion [MF-Linear]
RT : 0.70 min Scan# : (3,5)
BP : m/z 189.0000 Int. : 1599.98
Output m/z range : 10.0000 to 1150.4358 Cut Level : 0.00 %

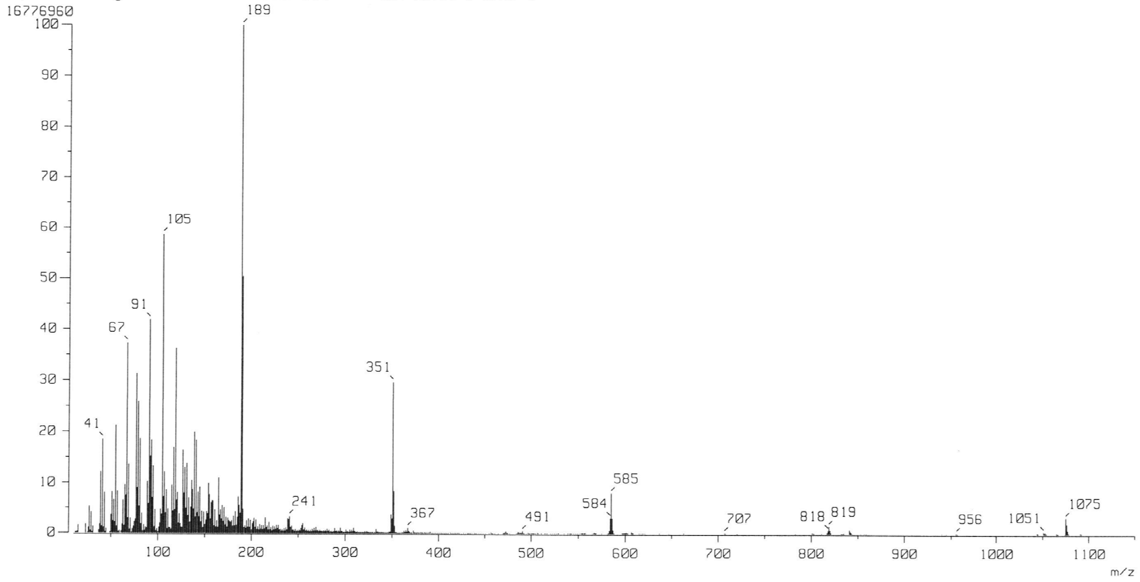


```
[ Elemental Composition ]                                   Page: 1
Data   : FAB-M166                  Date : 22-Jun-2015 17:41
Sample: HC001 E4
Note  : m-NBA
Inlet : Direct                     Ion Mode : FAB+
RT  : 0.90 min                     Scan#: (16,69)
Elements : C 150/0, H 200/0, O 10/0
Mass Tolerance       : 10ppm, 5mmu if m/z < 500, 10mmu if m/z > 1000
Unsaturation (U.S.) : -0.5 - 100.0

Observed m/z Int%   Err[ppm / mmu]    U.S.  Composition
  405.2642     4.6     +0.3 /  +0.1     6.5  C 24 H 37 O 5
```

```
[ Theoretical Ion Distribution ]                            Page: 1
Molecular Formula : C24 H37 O5
                       (m/z   405.2641, MW   405.5548, U.S.  6.5)
Base Peak :   405.2641, Averaged MW :    405.5509(a),    405.5517(w)

    m/z       INT.
  405.2641 100.0000 **********************************************************
  406.2675  26.8841 ****************
  407.2703   4.4677 ***
  408.2730   0.5541
  409.2756   0.0554
  410.2783   0.0047
  411.2809   0.0003
```

Figure S11. HR-FAB-MS of 15-*epi*-dihydromacrolactin F (**2**)

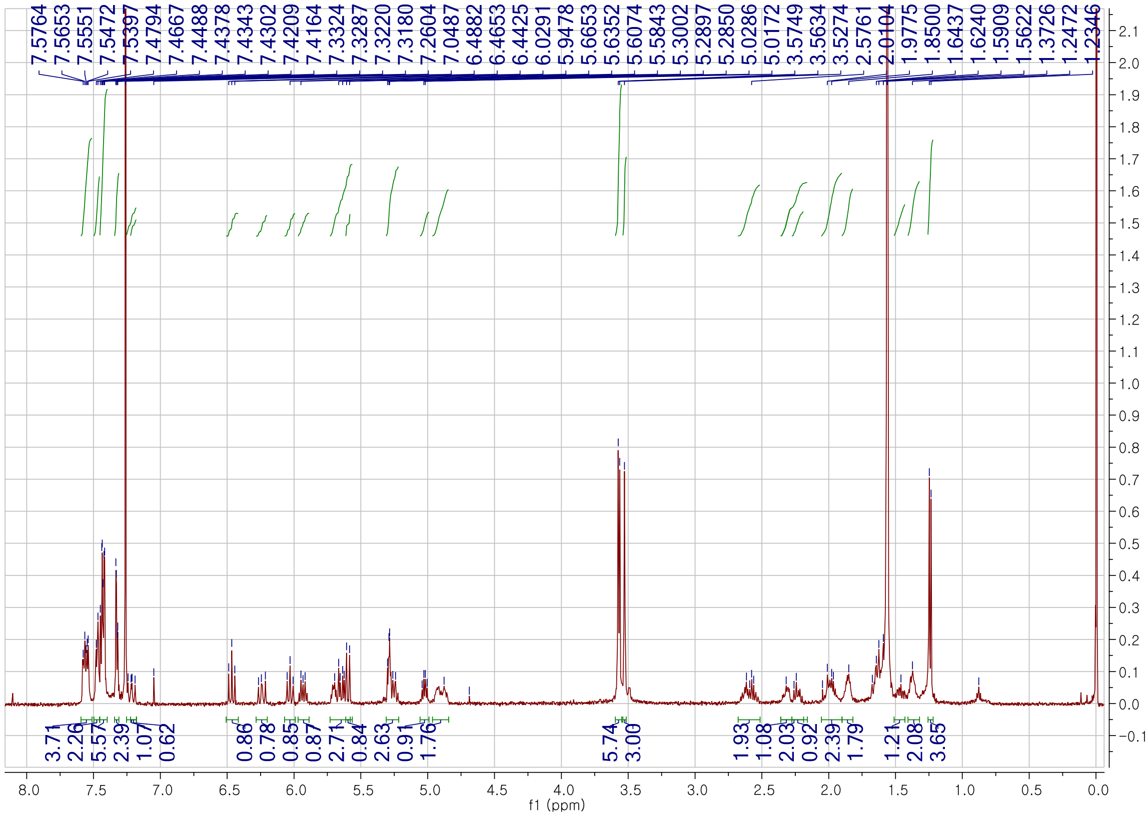

Figure S12. ^1H NMR (500 MHz, $CDCl_3$) spectrum of *tris*-(*S*)-MTPA *e*ster of **2** (**2a**)

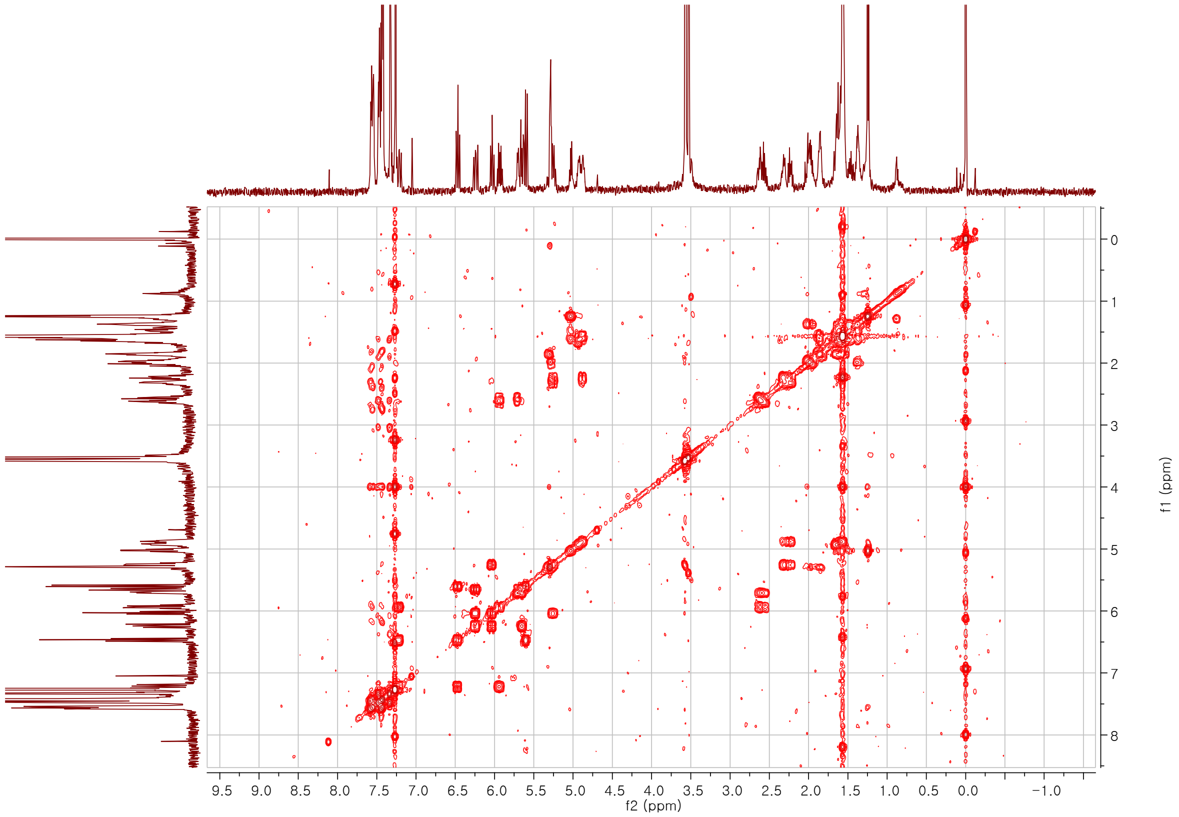

Figure S13. ^1H-^1H COSY (500 MHz, $CDCl_3$) spectrum of *tris*-(*S*)-MTPA *e*ster of **2** (**2a**)

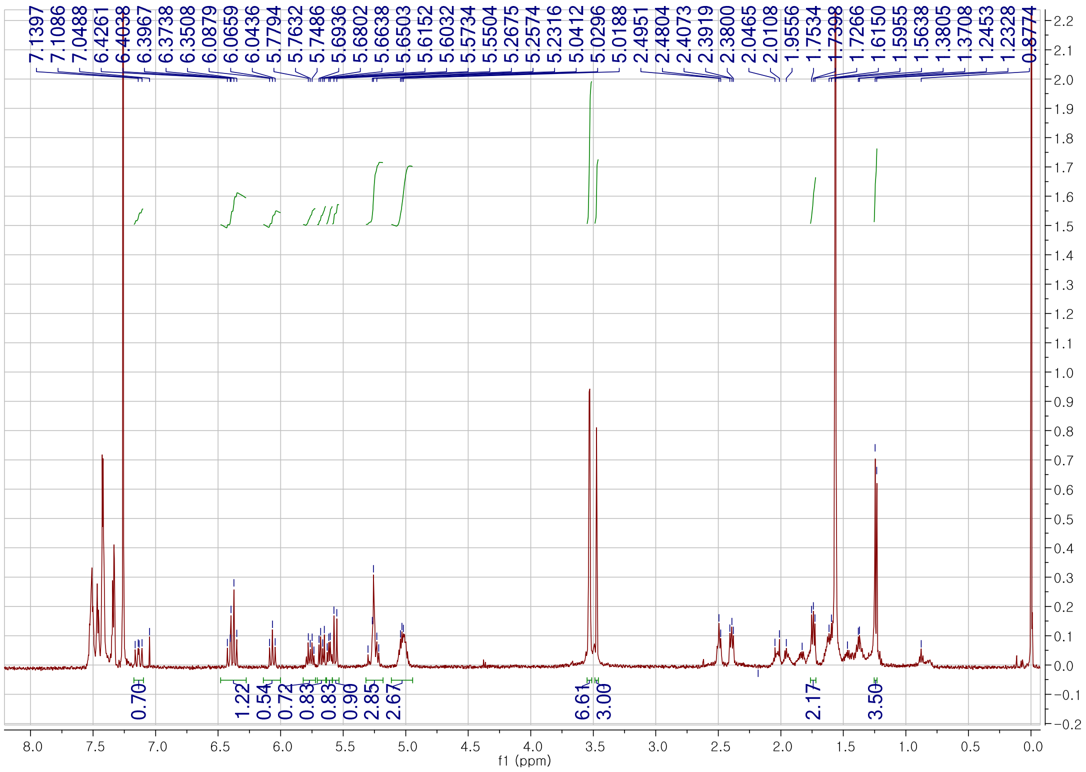

Figure S14. ^{1}H NMR (500 MHz, $CDCl_3$) spectrum of *tris*-(*R*)-MTPA *e*ster of **2** (**2b**)

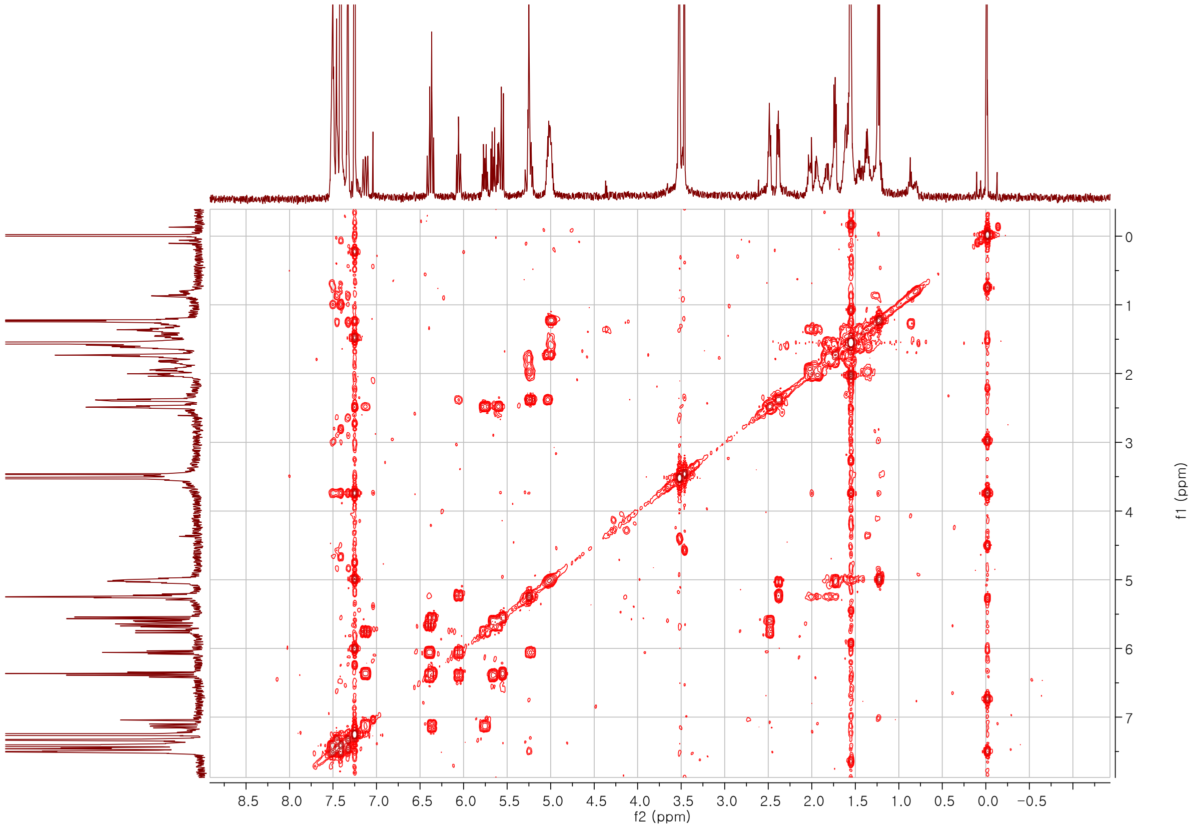

Figure S15. ^{1}H-^{1}H COSY (500 MHz, $CDCl_3$) spectrum of *tris*-(*R*)-MTPA *e*ster of **2** (**2b**)

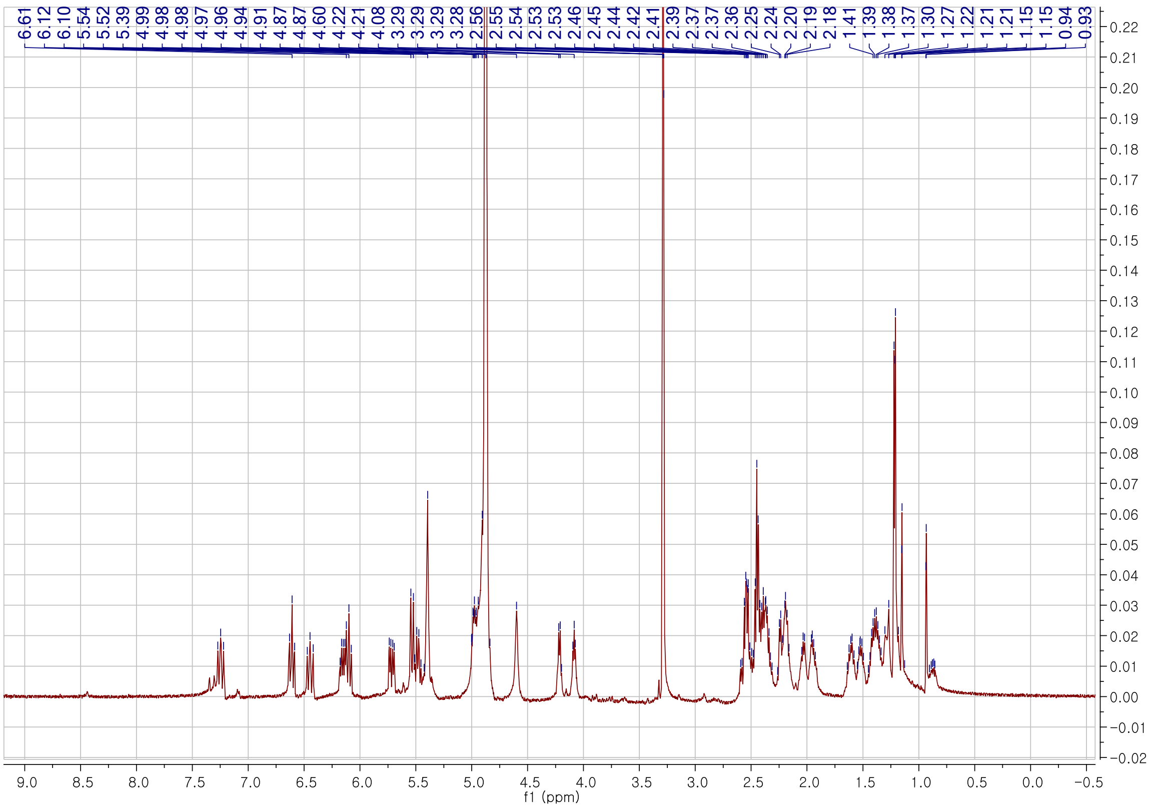

Figure S16. ^{1}H NMR (500 MHz, CD_3OD) spectrum of macrolactin F (**3**)

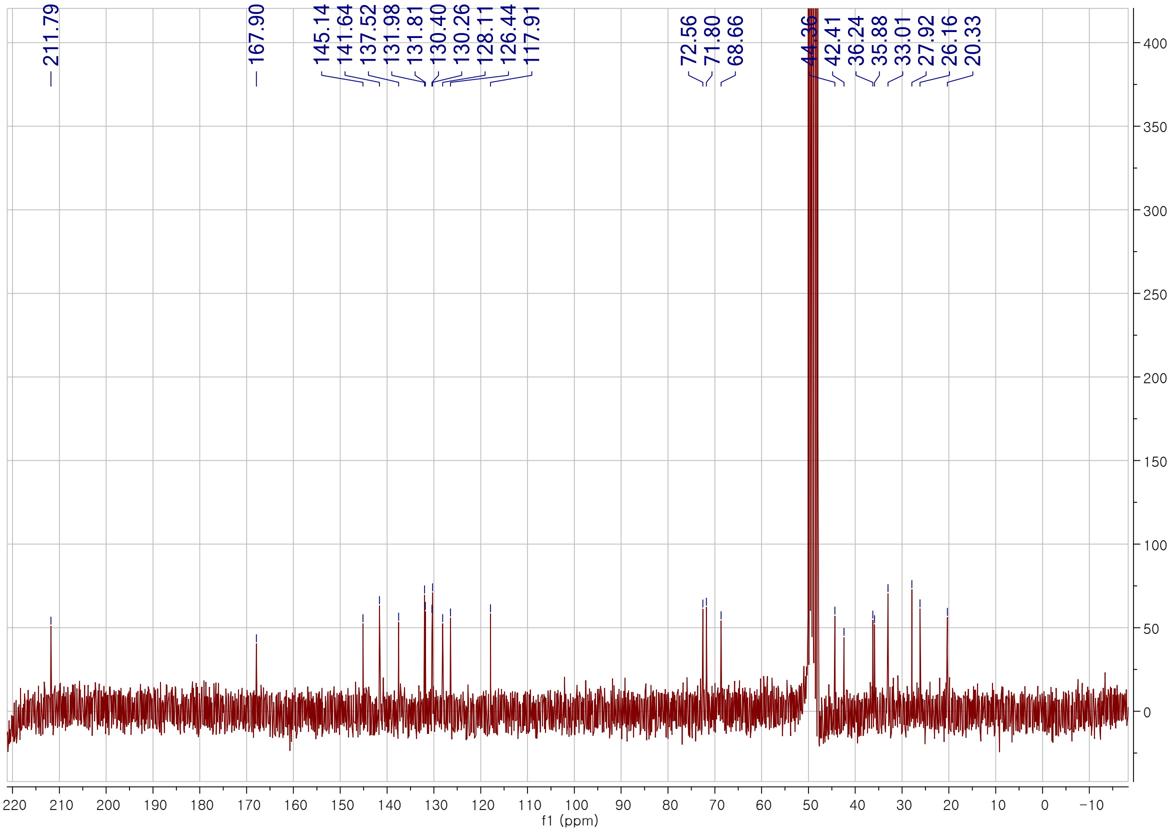

Figure S17. ^{13}C NMR (125 MHz, CD_3OD) spectrum of macrolactin F (**3**)

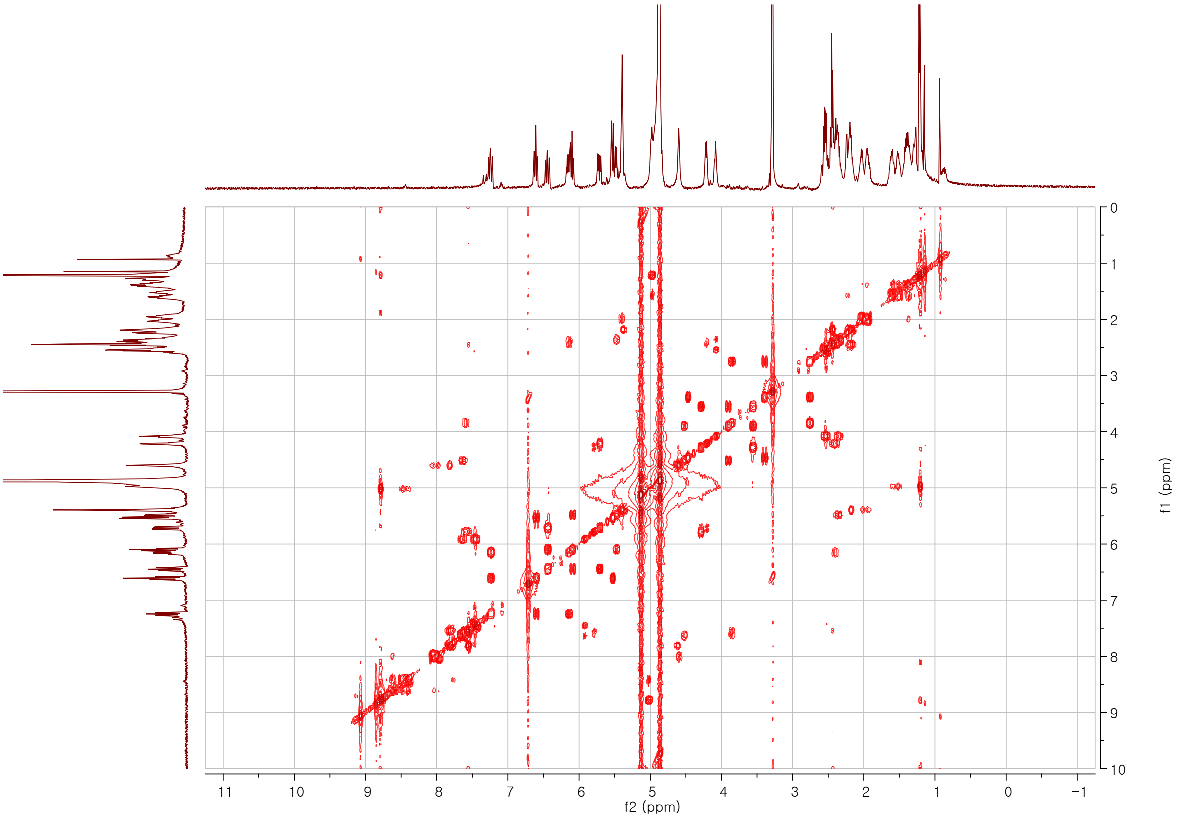

Figure S18. ^{1}H-^{1}H COSY (500 MHz, CD_3OD) spectrum of macrolactin F (**3**)

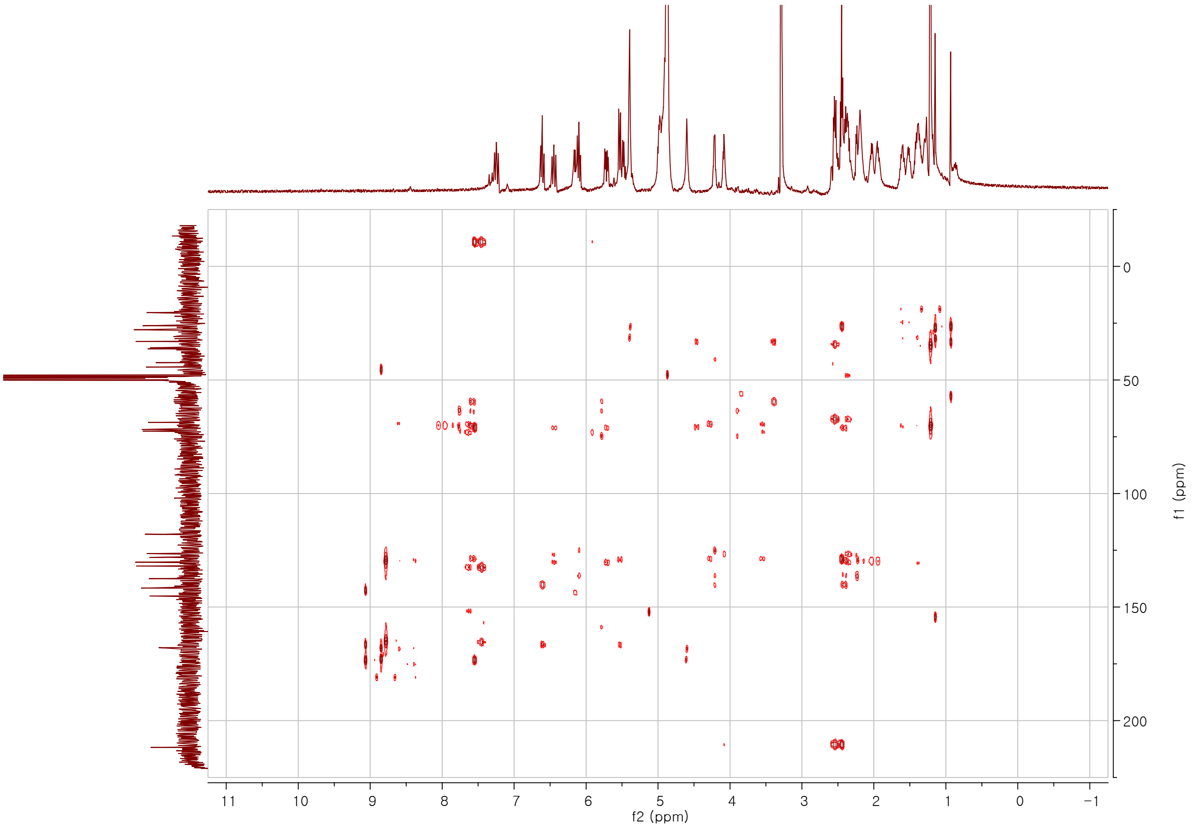

Figure S19. HMQC (500 MHz, CD_3OD) spectrum of macrolactin F (**3**)

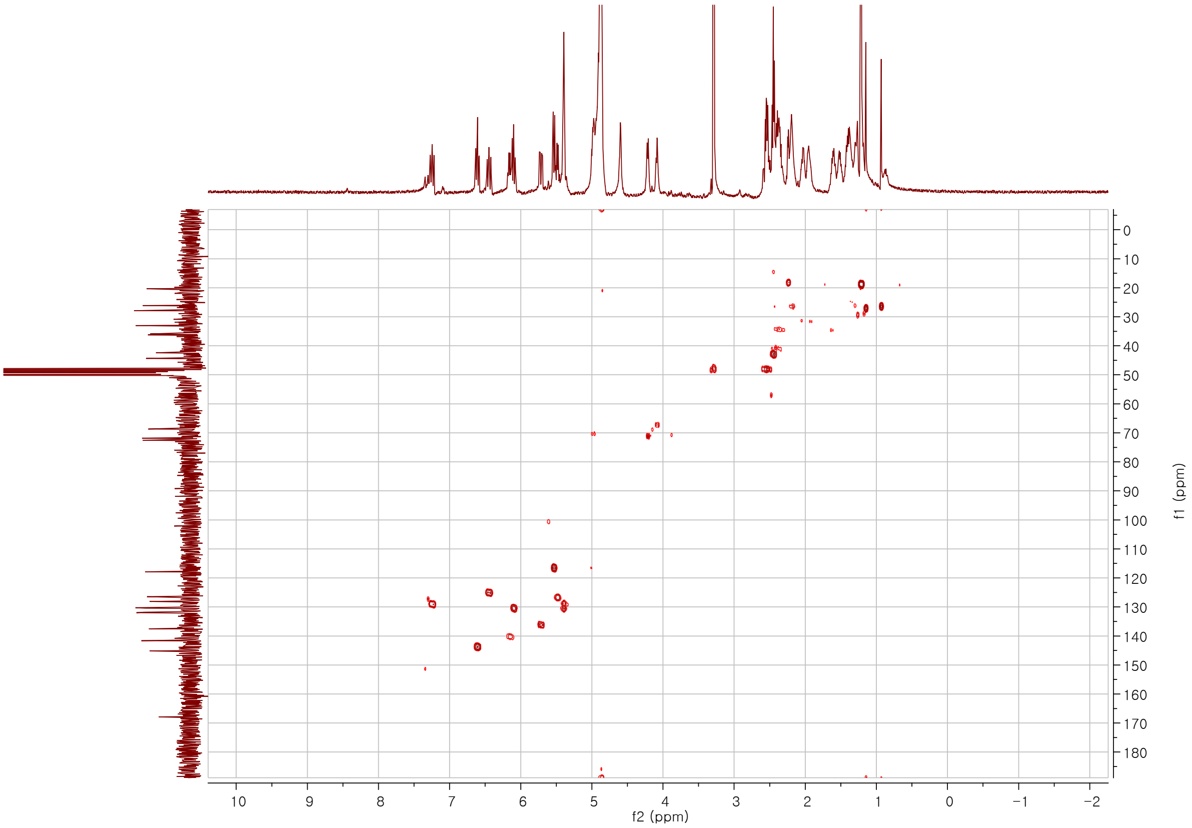

Figure S20. HMBC (500 MHz, CD_3OD) spectrum of macrolactin F (**3**)
